# *Helicobacter pylori* infection aggravates hepatic steatosis by lactylation-driven WTAP-mediated m^6^A modification

**DOI:** 10.1080/19490976.2025.2599543

**Published:** 2025-12-12

**Authors:** Han Chen, Zi Wang, Yan Wang, Shuo Li, Wei Su, Yuting Shao, Guoxin Zhang, Yun Liu, Qiang Ye, Xiaoying Zhou

**Affiliations:** a Department of Gastroenterology, The First Affiliated Hospital with Nanjing Medical University, Nanjing, Jiangsu, People's Republic of China; b The First Clinical Medical College, Nanjing Medical University, Nanjing, People's Republic of China; c Department of Geriatrics, The First Affiliated Hospital with Nanjing Medical University, Nanjing, Jiangsu, People's Republic of China; d Department of Medical Informatics, School of Biomedical Engineering and Informatics, Nanjing Medical University, Nanjing, Jiangsu, People's Republic of China; e Department of Gastroenterology, Zhenjiang First People's Hospital, Zhenjiang, People's Republic of China

**Keywords:** *Helicobacter pylori*, hepatic steatosis, m^6^A, lactylation, WTAP

## Abstract

*Helicobacter pylori (H. pylori)* infection has been investigated as a potential risk factor for extragastric diseases, including metabolic dysfunction-associated fatty liver disease (MASLD). However, details of the underlying mechanisms remain inadequately understood. In this study, we elucidate that *H. pylori* infection exacerbates hepatic metabolic disorders both in vitro and in vivo, manifesting as increased lipid deposition and insulin resistance. Mechanistically, *H. pylori* infection upregulates hepatic m^6^A content, particularly increasing the expression of WTAP. Overexpression of hepatic WTAP promotes liver steatosis characteristics, including increased lipogenesis and decreased fatty acid oxidation (FAO) and oxidative phosphorylation (OXPHOS). Conversely, knockdown of hepatic WTAP mitigated hepato-steatosis and insulin resistance in high-fat diet (HFD) mice and hepatic cells. After *H. pylori* infection, lactate accumulates in the liver, which potently induces WTAP upregulation in HepG2 cells via H3K18 lactylation. Notably, we identified two lactylation modification sites, K99 and K134, on WTAP, which are essential for WTAP to regulate GLUT3 mRNA stability in an m^6^A-YTHDF1-dependent manner. The upregulation of GLUT3 subsequently enhanced glycolysis, establishing a feedback loop that resulted in increased lactate accumulation. In conclusion, our findings highlight the significance of lactylation-driven WTAP-mediated RNA m^6^A modification in the aggravation of hepatic steatosis due to *H. pylori* infection. Therefore, the status of *H. pylori* should be taken into account in MASLD treatment strategies. Furthermore, the WTAP–YTHDF1–GLUT3 axis may be a potentially promising therapeutic target for MASLD progression.

## Introduction

Metabolic dysfunction-associated fatty liver disease (MASLD) has emerged as the leading cause of chronic liver diseases, with current estimates suggesting that it affects as much as 38% of adults worldwide.^1^ MASLD is a complex disorder affecting multiple systems, wherein systemic insulin resistance and associated metabolic dysfunction are pivotal in its development.^2^^,^^3^ Recent studies unveiled mechanisms referred to as ‘multiple hit’ pathways, indicating that uncomplicated steatosis can progress to MASLD and even to hepatocellular carcinoma (HCC) due to several contributing factors, including mitochondrial dysfunction, oxidative stress, insulin resistance, and alterations in microbiota composition or bacterial infection.^4^

*Helicobacter pylori* (*H. pylori*) is a gram-negative bacterium that infects nearly half of the global population, leading to a range of gastrointestinal disorders,^5^^,^^6^ as well as multiple extra-gastrointestinal conditions, including insulin resistance, dyslipidemia, and obesity.^7-9^ Recently, the relationship between *H. pylori* infection and the risk of MASLD has been examined globally.^10-13^ Increasing evidence indicates that *H. pylori* infection is associated with a heightened risk of both prevalent and incident MASLD, particularly among middle-aged individuals across various countries.^10^^,^^11^ Patients with MASLD, especially advanced metabolic dysfunction-associated steatohepatitis (MASH), have an increased risk of *H. pylori* infection.^12^^,^^13^ However, it remains unclear whether *H. pylori* infection would in turn influence the pathogenesis of MASLD, and the underlying pathogenic mechanisms by which *H. pylori* accelerates the progression of MASLD are still inadequately understood. While *H. pylori* colonizes gastric epithelial cells, it has the potential to induce biological changes in extra-gastric organs through the production of outer membrane vesicles (OMVs).^14^ OMVs are nanoscale entities expelled from *H. pylori* infection and these vesicles contain a variety of components, including enzymes, phospholipids, nucleic acids, toxins, and proteins that originate from the cytoplasm, membrane, and outer membrane.^15^ Various eukaryotic cells can internalize these extracellular vesicles through diverse endocytosis mechanisms, subsequently delivering multiple virulence factors straight into the cytoplasm of the host cell.^16^ Previous studies have demonstrated that OMVs from *H. pylori* can enter the bloodstream and contribute to the activation of hepatic stellate cells and the development of liver fibrosis in vitro.^17^

N^6^-methyladenosine (m^6^A) is involved in the pathogenesis of various inflammatory states, including autoimmunity, cardiometabolic diseases, and several types of cancer, by influencing processes such as RNA stability, splicing, translation, and degradation.^18^ The deposition of m^6^A marks is catalyzed by a group of enzymes known as m^6^A methyltransferases (writers), including METTL3, METTL14, and WTAP^19^ and erasers, including FTO and ALKBH5.^20^ Increasing evidence suggests a pathological involvement of aberrant m^6^A modification levels and the expression of its modulators in MASLD, indicating the important roles of m^6^A modification in MASLD progression.^21^ Given that *H. pylori* infection can also induce m^6^A modification,^22^ we hypothesized that m^6^A may serve as a potential link between *H. pylori* infection and the progression of MASLD.

In this study, we revealed the pathological role of the m^6^A methyltransferase WTAP in *H. pylori*-induced hepatic steatosis. We further mapped WTAP's regulatory networks in the liver and identified two critical lactylation sites essential for its RNA-binding ability.

## Materials and methods

### *H. pylori* strains and culture conditions

Two strains of *H. pylori* (SS1 and 26695), purchased from BioVector NTCC Inc (Beijing, China), were utilized in this study. They were cultured in an anaerobic incubator set at 37 °C for a duration of three days. The incubation environment consisted of 5% oxygen, 10% carbon dioxide, and 85% nitrogen. The culture medium used was a Columbia blood agar plate supplemented with brain heart infusion broth, 7% defibrinated sheep blood, and 0.1% antibiotics. *H. pylori* SS1 strain was used in murine studies, while *H. pylori* 26695 strain was employed for OMVs isolation in cellular studies.

### Animals

All animal procedures were approved by the Institutional Animal Care and Use Committee of Nanjing Medical University (IACUC-2207010) and followed NIH guidelines. Four-week-old male C57BL/6J mice (Vital River Laboratory Animal Technology, 219) were kept in specific pathogen-free conditions. All mice had unrestricted access to ultrapure water and were maintained at 22−25°C on a 12-hour light/dark cycle. MASLD mice were fed a high-fat diet (HFD; 60% kcal fat, D12492, Research Diets) for 16 weeks to induce the MASLD model.

### *H. pylori* infection and eradication

MASLD mice were divided randomly into HFD (*n* = 5/group) and HFD + *H. pylori* (HFDHP, *n* = 5/group). HFDHP mice received oral gavage of *H. pylori* SS1 strain (1 × 10^8^ CFU/mL in Brucella broth, 0.01 mL/g) every 72 h for 4 weeks. Fecal *H. pylori* antigen (HpSA) was tested at week 4 post-infection. All mice were euthanized at week 34 for sample collection. For eradication, HFDHP mice were randomized to control and treatment groups (*n* = 5/group). The treatment group received daily oral triple therapy (lansoprazole 0.01 mL/g, amoxicillin 0.01 mL/g, clarithromycin 0.005 mL/g) for 2 weeks. Eradication efficacy *H. pylori* eradication was evaluated by fecal *H. pylori* antigen testing.

### AAVs injection

AAV8 serotype was chosen for its high hepatocyte transduction efficiency in intravenous injection.^23^ The WTAP coding sequence was cloned into the liver specific TBG promoter and packaged into AAV8 serotype (AAV8-TGB-WTAP-fluc, Vigene Biosciences). 1 × 10^12^ genome copies/mL of AAV8-TBG-WTAP-fluc or AAV8-TBG-Blank-fluc was injected to mice (*n* = 5/group) via tail vein. For WTAP knockdown, mice were injected with AAV8-U6-shWTAP-fluc or AAV8-U6-shBlank-fluc by tail vein at 1 × 10^12^ genome copies/mL. Bioluminescence in vivo imaging was detected 10 minutes after intraperitoneal administration of D-Luciferin potassium salt (150 mg/kg, Beyotime, ST196) using IVIS Spectrum (Perkinelmer, USA).

### Cell culture and treatments

HepG2, L-02, and HEK293T cells were maintained in DMEM (Gibco, C11995500BT) supplemented with 10% FBS (Invitrogen, 12103 C), 100 U/mL penicillin, and 100 U/mL streptomycin at 37 °C with 5% CO₂. To establish the MASLD model, HepG2 and L-02 cells were treated with a 2:1 mixture of oleic acid (Sigma-Aldrich, 75090) and palmitic acid (Sigma-Aldrich, P9767) at a total concentration of 0.6 mM for 24 h. For actinomycin D treatment, HepG2 cells were cultured with 1 µg/mL actinomycin D (MCE, HY-17559) and harvested after 4 h and 8 h of incubation. For lactate treatment, cells were incubated with 25 mM L-lactate (Sigma-Aldrich, L1750) for 48 h.

### Measurement of biochemical parameters

The Oral Glucose Tolerance Test‌ (OGTT) was performed after an overnight fast from 7 p.m. to 7 a.m. Blood glucose levels of tail vein samples were analyzed using a glucose analyzer (OneTouch Ultra, Lifescan, Johnson & Johnson, Milpitas, CA) at 0, 30, 60, 90, and 120 min after oral administration of glucose (1g/kg). For the Insulin Tolerance Test (ITT), 2 U/kg insulin were given via intraperitoneal injection following a 4 h fast from 8 a.m. to 12 p.m. Blood glucose levels were monitored at 0, 30, 60, 90, and 120 min post-insulin injection. Mouse serum insulin concentrations were quantified using Ultra Sensitive Mouse Insulin ELIZA Kit (Crystal Chem Inc, 90080). The concentrations of serum or intracellular triglycerides (TG) (A111-1-1), total cholesterol (TC) (A110-1-1), serum low-density lipoprotein cholesterol (LDL) (A113-1-1), high-density lipoprotein cholesterol (HDL) (A112-1-1), ketone bodies (HT169-1-1), glycogen (A019-2-1), and lactate acid (A043-1-1) were assessed using commercial assay kits supplied by Nanjing Jiancheng Biotechnology Company.

### Histological analysis

Liver tissues were fixed with 4% paraformaldehyde and then embedded in paraffin. Sectioned the samples at 5 µm thickness and mounted on glass slides. After deparaffinization and rehydration, the sample was stained using a Hematoxylin and Eosin (H&E) commercial kit (Servicebio, G1076) or a Masson's trichrome kit (Sigma-Aldrich, HT15) according to the manufacturer's instructions. Whole-section images were captured at 200× magnification with Nikon Eclipse Ci-L microscope. The quantity of liver fibrosis was determined by the ratio of collagen surface area to total surface area using ImageJ's color threshold tool.

The Warth-Starry silver staining was conducted with the Spirochete Silver Staining Kit (Solarbio-G1940). Initially, the paraffin-embedded tissues were deparaffinized using xylene and ethanol gradient. The slides were then submerged in the Acid Silver Solution for staining in a 56 °C water bath for 60 minutes. The slides were subsequently re-stained with Warthin-Starry Stain Solution in a water bath at 56 °C until they became yellowish brown. The Nikon Eclipse microscope was used to capture all images.

### *H. pylori* OMVs isolation and characterization

OMVs were isolated from *H. pylori* 26695 strain broth culture. Briefly, 500 ml culture media were centrifuged at 15,000 g for 15 min at 4 °C to remove the bacteria pellet, and the supernatant was collected and filtered through a 0.45 µm filter. The sample was then ultracentrifuged at 150,000 g for 3 h at 4 °C (Beckman) to pelleted OMVs. Resuspend the OMVs pellet with 10 ml sterile distilled water and was stored at −80 °C until use. OMVs were characterized by transmission electron microscopy (TEM) (HITACHI, H-7650) to observe the morphology, and the nanoparticle tracking analysis (NTA) (ParticleMetrix, ZetaView) was used to determine the size. The concentration was measured using a BCA kit (Beyotime, P0009), and western blotting was performed to confirm the successful extraction of OMVs. The indicators included CD63, CD9, Alix, Calnexin, CagA, VacA, and UreaA.

### OMVs labeling and tracking

OMVs were incubated with 2 μL PKH67 dye (MCE, 257277-27-3) in cold PBS for 15 min. The labeled OMVs were then centrifuged at 150,000 g for 70 min to remove excessive dye. Recipient HepG2 cells were co-cultured with 1 μg labeled OMVs for 4 h, and the cells were then fixed with 4% paraformaldehyde and stained with DAPI (Beyotime, C1002). For in vivo tracking, 5 μg labeled OMVs (in 1 mL PBS) were administered to mice via gavage. After 72 h, liver tissues were collected and fixed. Images were acquired using confocal microscopy (OLYMPUS IX83-FV3000).

### Cell viability

Cell viability was assessed using the MTT assay (Beyotime, C0009S). Briefly, cells were seeded in 96-well plates (5 × 10 ³ cells/well) and treated with OMVs from *H. pylori* 26695 strain (1 μg) for 24 h. MTT reagent (10 μL/well) was added into the cell and incubated for 4 h at 37 °C. The reaction was stopped with DMSO, and absorbance was measured at 570 nm. The cell viability was calculated as: (OD_treated/OD_control) × 100%.

### Oil red O staining

HepG2 cells or liver sections were fixed with 4% paraformaldehyde for 15 min, followed by staining with filtered Oil Red O solution (Nanjing Jiancheng, D027-1-1) for 30 min at 37 °C. Samples were then rinsed with PBS and destained with 60% isopropanol, and the nuclei were counterstained with DAPI. After staining, lipid droplets were imaged using an Olympus BX53 microscope and the Oil Red O‑positive area was quantified in ImageJ as a percentage of positively stained area relative to the total.

### Lentivirus construction and transduction

The coding sequence of WTAP and mutant lacking H3-H4 domain (148-249a) were cloned into the third-generation lentivirus vector to generate pLVX-WTAP^WT^ or pLVX-WTAP^MU^. The primer sequences are presented in Supplementary Table 1. pCMV-Gag/Pol and pCMV-VSVG plasmids were co-transfected with 10 μg pLVX-WTAP^WT^ or pLVX-WTAP^MU^ into HEK293T cells at a ratio of 3:1:4 using Lipofectamine 3000 (Invitrogen, L3000001). The supernatant was collected 48 h after transfection, and centrifuged at 1,500 g for 15 min to remove cell debris. HEK293T cells were transduced with a serial dilution of the lentiviral particles in the presence of 8 µg/mL polybrene to test the optimal multiplicity of infection (MOI). HepG2 cells were infected with lentivirus at a MOI of 10 and subjected to subsequent analysis.

### Real-time quantitative PCR (RT-qPCR)

Total RNA was extracted from cells and liver tissues using TRIzol reagent (Invitrogen, 15596018CN) following the manufacturer's instructions. RNA concentration and purity were measured using a NanoDrop ND-1000 spectrophotometer. cDNA was synthesized from 1 µg of total RNA using the PrimeScript RT Reagent Kit (Takara, RR047Q). qPCR was performed on a LightCycler® 96 System (ROCHE) with SYBR Green Master Mix (Servicebio, G3323-01) under the following conditions: 94 °C for 5 min; 40 cycles of 95 °C for 15 s and 60 °C for 60 s. Gene expression was normalized to *β*-actin and calculated using the 2–ΔΔCq method. All primer sequences are listed in Table S1.

### Immunofluorescence staining

All cells were fixed with 4% paraformaldehyde at room temperature. Tissue sections were deparaffinized, rehydrated, and subjected to antigen retrieval using Tris-EDTA buffer (pH 9.0) (Beyotime, R0225). Samples were then incubated with primary antibodies, including anti-F4/80 antibodies (Servicebio, GB113373; 1:200), anti-WTAP antibodies (Proteintech, 10200-1-AP; 1:200), anti-H3K18la-antibodies (PTM-bio, PTM-1427RM; 1:200), anti-Pan-Kla-antibodies (PTM-bio, PTM-1401RM; 1:200) overnight at 4 °C. Washed the samples three times with PBS and incubated with CoraLite488/594-conjugated secondary antibodies (Proteintech, SA00013-2, SA00013-1, SA00013-4, SA00013-3; 1:200) for 2 h at room temperature. Nuclei were stained with DAPI for 10 min. All images were acquired using a laser scanning confocal microscope. Quantification was performed based on mean gray values using Image J software.

### Dot blot analysis of m^6^A

The mRNA was purified using the Dynabeads mRNA Purification Kit (Invitrogen, 61006), and then denatured at 95 °C for 5 min, followed by chilling on ice. 400-800 ng mRNA was spotted onto a positively charged nylon membrane (GE Healthcare) and air-dried for 5 min. UV-crosslinked (254 nm) the membrane and blocked with 5% nonfat milk for 45 min. After blocking, incubate the membrane with anti-m^6^A antibody (Synaptic Systems, 202003, 1:1000) overnight at 4 °C. Wash the membrane three times, and incubate with HRP-conjugated secondary antibody (Proteintech, SA00001-2, 1:5000) for 1 h at room temperature. The membrane was developed using an enhanced chemiluminescent substrate (Beyotime, P0018S). Equal loading was confirmed by methylene blue staining (0.02%; MCE, HY-14536). Dot intensity was quantified with ImageJ software.

### Quantification of m^6^A level

Total RNA m^6^A methylation was quantified using the EpiQuik m^6^A RNA Methylation Quantification Kit (Epigentek, *P*-9005-48) according to the manufacturer's protocol. In brief, 400 ng of RNA or control were immobilized onto the assay wells. The wells were then incubated with m^6^A capture antibodies for 90 min at 37 °C, followed by another incubation with the detection antibody for 30 min at room temperature. After the incubation with developer solution and stop solution, the absorbance was measured at 450 nm using a Synergy 4 microplate reader (Agilent BioTek).

### Western blot

Cells or mouse tissues were lysed using lysis buffer (Beyotime, P0013) with protease inhibitor cocktail (Sigma-Aldrich, P8340). Nuclear and cytoplasmic proteins were isolated with Nuclear/Cytosolic Fractionation kit (BioVision, 266-100) following the manufacturer's instructions. Proteins were separated by SDS-PAGE and transferred to PVDF membranes (Sigma-Aldrich, HVLP02500). Blocked the membranes with 5% non-fat milk and then incubated with primary antibodies at 4 °C overnight (detailed in Table S2), followed by HRP-conjugated secondary antibodies at 1:10,000 dilution for 1 h at room temperature. Signals were detected using a Tanon-5200Multi imaging system, and band intensity was quantified with ImageJ.

### Detection of reactive oxygen species (ROS) levels

ROS levels were detected using the DCFH-DA probe (Beyotime, S0033S). Cells were seeded in 6-well plates at 1.0 × 10⁵ cells/well. For tissue samples, single-cell suspensions were prepared prior to incubation with 10 μM DCFH-DA for 30 min at 37 °C. After washing with PBS, fluorescence intensity was measured by flow cytometry (BD FACSCalibur, USA).

### Oxygen consumption rate (OCR) and extracellular acidification rate (ECAR)

OCR and ECAR were measured using a Seahorse XFe 96 Analyzer (Seahorse Bioscience) with the XF Cell Mito Stress Test Kit (Agilent Technologies, 103015-100 and 103595-100). In brief, HepG2 cells were seeded at 2 × 10⁴ cells/well in a 96-well plate 24 h before the OMVs treatment. After 48 h incubation, baseline OCR and ECAR was measured. The oligomycin (1 mM), FCCP (2 mM), and a combination of rotenone and antimycin A (0.5 mM) were injected into each well for OCR measurement. Glucose (10 mM), oligomycin (1.5 μM), and 2-DG (50 mM) were added for ECAR analysis. Three cycles of 2 min mix, 2 min wait and 3 min measurement were used to measure the OCR and ECAR. Data were analyzed using Seahorse Wave software.

### Lactate detection

To assess the intracellular lactate level, pre-treated cells were harvested and lysed with lysis buffer. Cell lysates were deproteinized using a TCA-based preparation kit (Abcam, ab204708) to eliminate endogenous LDH activity, and lactate levels were determined using the L-Lactate Assay Kit (Beyotime, S0208S). Absorbance was measured at 570 nm using a Synergy 4 microplate reader, and lactate concentrations were calculated based on the standard curve and normalized to cell number.

### MeRIP-qPCR

m^6^A modifications were detected using the Ribo MeRIP m^6^A Transcriptome Profiling Kit (RiboBio, C11051-1). Briefly, 100 μg of total RNA was fragmented, and 10 μg of the fragmented RNA was reserved as the input control. Immunoprecipitated the remaining RNA with 5 μg of anti-m^6^A antibody conjugated magnetic beads for 2 h at 4 °C. After washing, the bound mRNA was eluted and purified. The enriched RNA was subsequently analyzed by qPCR analysis and normalized to input as:%Input = 2^(Ct[input] - Ct[IP]) × 10. Primers sequences are listed in Table S1.

### Chromatin immunoprecipitation (ChIP)-qPCR

The SimpleChIP® Enzymatic Chromatin IP Kit (Cell Signaling Technology, 9003) was used to perform the ChIP assay following the manufacturer's instructions. Cells were crosslinked with 1% formaldehyde and lysed with lysis buffer. The sample was then digested with micrococcal nuclease at 37 °C for 20 min and stopped by the addition of 0.5 M EDTA. After sonication, a total of 10 μg DNA fragment was reserved as the input control, and the remaining fragment was subsequently incubated with 2 μg of anti-H3K18la antibody or anti-IgG antibody overnight at 4 °C. After washing, the enrichment of H3K18la bound DNA was then eluted and extracted for qPCR analysis, and Ct values were used to calculate the percentage of input enrichment. Primers sequences are listed in Table S1.

### Co-immunoprecipitation (Co-IP) assay

Co-IP was performed using a Pierce Classic Magnetic IP/Co-IP Kit (88,804, Thermo Fisher Scientific). Cells were lysed with cold lysis buffer and supernatant was collected. Approximately 1000 μg protein was incubated with specific IP antibody (1:50) or IgG (1:50) at 4 °C on a rotating platform overnight. Pierce Protein A/G Magnetic Beads (25 μL) were added to the antigen sample/antibody mixture and incubated at room temperature for 1 h. After washing, the target antigen–antibody complex was eluted with 100 μL of elution buffer and 10 μL of neutralization buffer, followed by western blotting analysis.

### Lactylation detection

Lactylation of WTAP was detected by LC-MS in HepG2 cells transfected with pLVX-WTAP and treated with 25 mM L-lactate for 48 h. Cell lysates were prepared with 1% SDS and subjected to immunoprecipitation using an anti-WTAP antibody. Proteins were separated by SDS-PAGE and stained with coomassie blue. The target bands were excised and the subsequent mass spectrometry analysis was performed by BGI (Shenzhen) using an LTQ Orbitrap Elite instrument (Thermo Fisher Scientific).

### Molecular docking

The 3D structure of WTAP was predicted by AlphaFold tool and the structure of L-Lactate was downloaded from PubChem (CID:5460161). The binding of L-Lactate and WTAP was simulated using Auto-Dock 4.0. WTAP was placed into the substrate binding site of L-Lactate as the start point of docking. The conformation with the lowest binding energy of WTAP was considered as the L-Lactate-bound conformation.

### Luciferase reporter assay

The wild-type GLUT3 or mutant and 3′UTR (nt 7919280–7919500) were cloned into the pGL3-control firefly luciferase vector (Vazyme, DL101). Specifically, the mutant reporterswere generated by substituting adenosine at position 1915aa and 2116aa with thymidines in the m6A motifs. 0.5 μg reporter plasmid and 25 ng pRL-TK renilla luciferase vector (internal control) were co-transfected to EK293T cells using jetPRIME (Polyplus, 101000046). After 48 h, luciferase activity was measured using the Dual-Glo system (Promega) and normalized to renilla luciferase activities. All transfections were performed in triplicate.

### RNA immunoprecipitation (RIP) assays

RIP assays were performed using the Magna RIP™ Kit (Millipore, 17-700) according to the manufacturers' recommendations. Specifically, the harvested cells were lysed in RIP lysis buffer. Collected the cell lysate and reserved 10 μg as input control. The remaining sample was incubated with 5 μg anti-YTHDF1 (Proteintech, 26787-1-AP) or IgG (Millipore, I5381) conjugated beads overnight at 4℃, respectively. After extensively washing, the complexes were eluted and decrosslinked with proteinase K. Purified RNA was reverse transcribed to synthesize the cDNA with PrimeScript RT Reagent Kit (Takara, RR047Q) and analyzed by qPCR. The enrichment of target RNAs was calculated using the ΔΔCt method, normalized to the input control and compared to the IgG background signal. Primers sequences are listed in Table S1.

### RNA decay assay

HepG2 cells were transfected with 50 nM of si-YTHDF1 or si-NC for 48 h (Target sequence was shown in Table S1), followed by the treatment of 5 μg/mL actinomycin D (MedChemExpress, HY-17559). Harvested the cells at 0, 4, and 8 h post-treatment and purified the RNA and reverse-transcribed into cDNA. The mRNA levels of GLUT3 were quantified by qPCR using primers listed in Table S1. The mRNA half-life was estimated by performing linear regression analysis on the remaining mRNA percentage plotted against time.

### Metabolomic analysis and data processing

Harvested the liver tissue and ground in liquid nitrogen to extract the metabolites. Ultraperformance liquid chromatography-tandem mass spectrometry (UHPLC-MS/MS) analyzes were executed using a Vanquish UHPLC System (Thermo Fisher, Germany) in conjunction with an Orbitrap Q Exactive TM HF mass spectrometer (Thermo Fisher, Germany), facilitated by Novogene Co., Ltd (China). Chromatographic separation was achieved on a Hypersil Gold column (100 × 2.1 mm, 1.9 μm) under a 17-min linear gradient at a flow rate of 0.2 mL/min. One alipuot was analyzed using positive ion conditions and was eluted from a T3 column (Waters ACQUITY Premier HSS T3 Column 1.8 µm, 2.1 mm × 100 mm) using 0.1% formic acid in water as solvent A and 0.1% formic acid in acetonitrile as solvent B in the following gradient: 5 to 20% in 2 min, increased to 60% in the following 3 mins, increased to 99% in 1 min and held for 1.5 min, then came back to 5% mobile phase B within 0.1 min, held for 2.4 min. The Q Exactive™ HF mass spectrometer was operated in both positive and negative polarity modes, with an applied voltage of 3.5 kV, a capillary temperature of 320 °C, a sheath gas flow rate of 35 psi, an auxiliary gas flow rate of 10 L/min, an S-lens RF level set to 60, and an auxiliary gas heater temperature of 350 °C.

Raw data were subsequently processed utilizing Compound Discoverer 3.1 (CD3.1, Thermo Fisher) for peak alignment, peak picking, and quantification of each metabolite. Normalization of peak intensities and predict the molecular formula based on additive ions, molecular ion peaks and fragment ions. Subsequently, the peaks were matched against the McCloud, mzVault, and MassList online databases. For the analysis of two groups, differential metabolites were identified based on Variable Importance in Projection (VIP) scores greater than 1, and *P*-values less than 0.05, as determined by Student's t-test. VIP scores were derived from the results of Orthogonal Partial Least Squares Discriminant Analysis (OPLS-DA), which also included score plots and permutation plots. These analyzes were conducted using the R (version 3.4.3) package MetaboAnalystR. Prior to performing OPLS-DA, the data underwent log transformation (log2) and mean centering. To mitigate the risk of overfitting, a permutation test with 200 permutations was conducted. The identified metabolites were annotated with the Kyoto Encyclopedia of Genes and Genomes (KEGG) database.

### MeRIP-Seq and dataanalysis

The preparation of mRNAs fragments was as described above, and save 5 µg as input control for RNA sequencing. Incubated 500 µg fragmented mRNAs with 5 µg anti-m^6^A polyclonal antibody (Synaptic Systems, 202003) in IP buffer (150 mM NaCl, 0.1% NP-40, 10 mM Tris-HCl, pH 7.4) for 2 h at 4 °C. Subsequently, the m^6^A antibody precipitated mixture was incubated with protein-A beads for additional 2 h at 4 °C. After washing, the bound mRNA was eluted using N^6^-methyladenosine (Berry & Associates, PR3732) in IP buffer and purified with Trizol. The mRNAs were then employed for RNA sequencing library preparation using the NEBNext® Ultra™ RNA Library Prep Kit (NEB, E7530L) according to the manufacturer's instructions. The input and m^6^A immunoprecipitated sample were subjected to 150 bp paired-end sequencing on an Illumina HiSeq platform.

Paired-end reads were then subjected to quality control using a Q30 threshold. Following the trimming of 3' adapters and the removal of low-quality reads using Cutadapt software (v1.9.3), the reads were aligned to the reference genome (UCSC HG19) utilizing Hisat2 software (v2.0.4). Methylated sites on RNAs, referred to as peaks, were identified using MACS software. Differentially methylated sites on RNAs were detected with diffReps (|Log2FC| > 2, FDR < 0.01). The identified peaks were subsequently mapped to the transcriptome using custom scripts (Tables S3−S5).

### Statistical analysis

All statistical analyzes were performed using GraphPad Prism 9.0. Normality was assessed using Shapiro-Wilk and Kolmogorov-Smirnov tests, and a *p*-value < 0.05 indicated normal distribution. For comparisons, a two-tailed t-test was used for normally distributed data, and the Mann-Whitney U test for non-normal data. Multiple-group comparisons employed one-way ANOVA with Bonferroni's post hoc test (normal distribution) or Kruskal-Wallis test (non-normal). Data are presented as mean ± SD (normal) or median with interquartile range (non-normal). A *p*-value < 0.05 was considered significant, with *p < 0.05, **p < 0.01, ***p < 0.001, and “ns” for not significant.

## Results

### *H. pylori* infection aggravates hepatic steatosis in HFD mice

**Figure 1.**
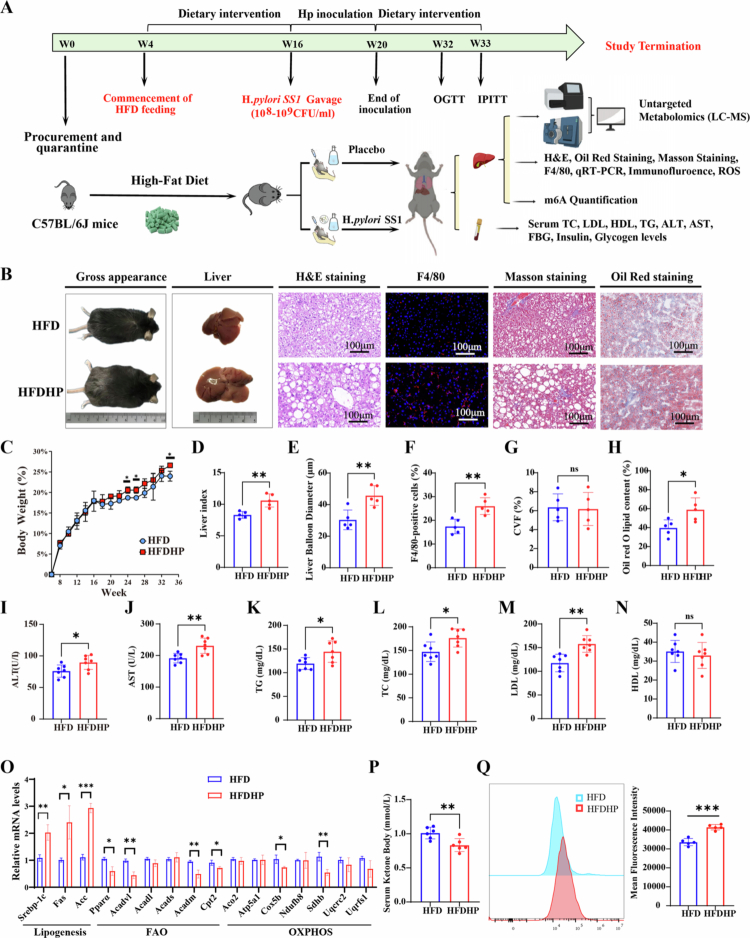
*H. pylori* infection aggravates hepatic steatosis in C57BL/6 mice with HFD diets. A) Schematic diagram of the animal experiment, *n* = 5 per group, mice from HFDHP group were infected with *H. pylori SS1 strain*; B) Representative images of gross appearance of mice, the liver histology (1 cm) and photomicrographs of fixed liver sections after staining with H&E (100μm), F4/80 antibody (100μm), Masson staining (100μm), and ORO (100μm); C) Percentage of weight change (%) from 6 to 34 weeks; D) Quantification of the liver index; E) Average liver balloon diameter (μm) of each group; F) F4/80 positive cells (%) of each group; G) CVF% of each group. H) ORO lipid content (%) of each group; I-*N*) Serum concentration of biomedical indexes, including ALT (U/L), AST (U/L), TG (mg/dL), TC (mg/dL), LDL (mg/dL), and HDL (mg/dL); O) Quantitative PCR data of genes involved in lipogenesis, FAO and OXPHOS; *P*) Serum ketone body levels of each group; Q) Representative histograms and mean fluorescence intensity of ROS production assessed by flow cytometry. Statistical analysis was performed using Student's *t*-test (two groups) and one-way analysis of variance (ANOVA) (multiple groups) followed by Bonferroni's test. *p < 0.05; **p < 0.01; and ***p < 0.001.

HFD mice with or without *H. pylori* SS1 strain infection was established, as illustrated in Figure 1A. The validation of *H. pylori* inoculation, both in vitro and in vivo, was presented in Figure S1. Body weight change and liver index of *H. pylori*-infected HFD mice were significantly higher at the 34th week, as compared to their negative counterparts (Figure 1B-D). Liver H&E staining revealed notable microvesicular steatosis with larger balloon diameters in infected mice, indicating that *H. pylori* infection aggravated hepatic steatosis in fatty mice. Although collagen volume fraction and *α*-SMA staining, indicating hepatic fibrosis, showed no significant difference, there was a marked increase in the number of infiltrating macrophages (F4/80-positive cells) in *H. pylori-*infected mice (Figures 1B-H, S2A), suggesting more severe hepatic lobular inflammation after *H. pylori* infection. Furthermore, we observed that *H. pylori*-infected mice exhibited elevated serum levels of ALT, AST, TC, TG, and LDL, while HDL levels showed no statistically significant difference (Figure 1I-N). Besides, *H. pylori* infection suppressed the expression of genes involved in FAO (*Pparα*, *Acadvl*, *Acadm* and *Cpt2*) and OXPHOS (*Cox5b*, *Sdhb*), while enhanced the expression of genes associated with de novo lipogenesis (*Srebp-1c*, *Fas*, *Acc*) (Figure 1O). Additionally, the inhibition of fatty acid oxidation due to *H. pylori* infection resulted in decreased serum levels of ketone bodies, which are closely linked to abnormal lipid deposition (Figure 1P). Given oxidative stress is an index indicating the severity of hepatic steatosis, we found by FACS analysis that a significant increase in ROS levels was observed after *H. pylori* infection (Figure 1Q). Significant increases in fasting blood glucose and insulin levels were observed, along with a decrease in glucagon levels in infected mice (Figure S2B-C). Besides, glucose intolerance and insulin sensitivity were substantially impaired (Figure S2D-E). These results confirmed that *H. pylori* infection plays a critical role in aggravating hepatic steatosis in vivo.

### *H. pylori* infection dysregulates lipid metabolism in vitro through OMVs

**Figure 2.**
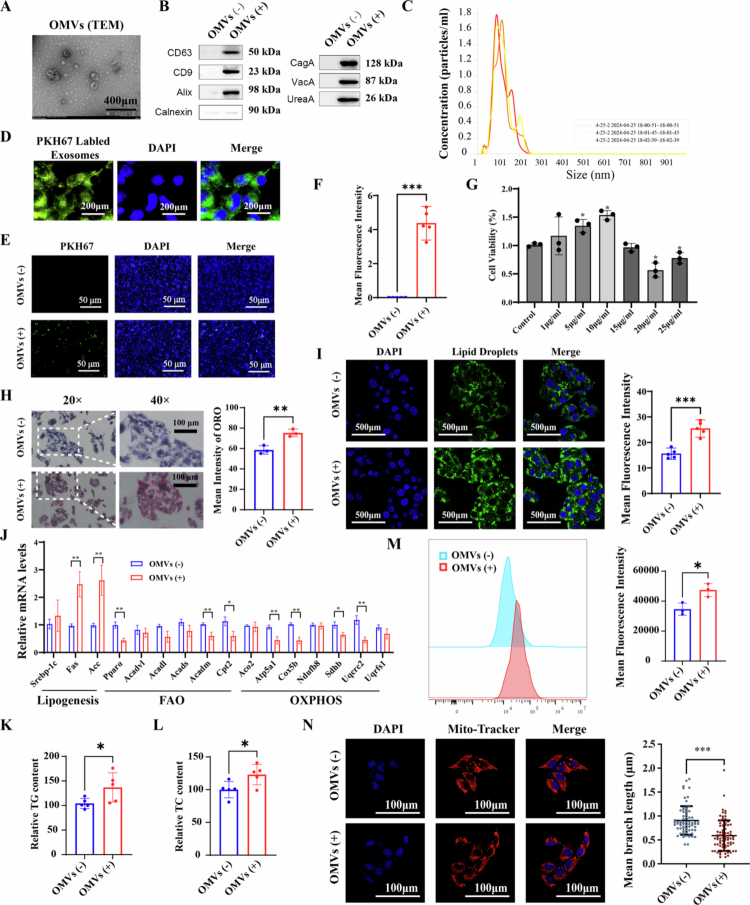
*H. pylori* infection dysregulates lipid metabolism in vitro through OMVs. A) Representative images of OMVs isolated from *H. pylori* 26695 strain identification by transmission electron microscope (400μm); B) Components of *H. pylori* OMVs identification by Western blotting; C) NTA shows the sizes and total concentration of OMVs; D) immunofluorescence images (200μm) of *H. pylori* OMVs internalized into HepG2 cells: The green signals represent PKH67 labeled OMVs, while the blue represents DAPI; E-F) Representative images of immunofluorescence with mean fluorescence intensity of liver tissues (50μm) in C57/BL6 mice with or without *H. pylori* OMVs gavage. Left: Signal for PKH67 labeled OMVs (green). Middle: DAPI counterstain (blue) identifying cell nuclei. Right: Merged image; G) Cell viability (%) of HepG2 cells co-cultured with OMVs of different concentrations using CCK8 analysis, *n* = 3 per group; H) Representative images and mean intensity of ORO staining of HepG2 cells with or without *H. pylori* OMVs; I) Immunofluorescence of lipid droplets of HepG2 cells with or without *H. pylori* OMVs: the green signals represent lipid lipid droplets, while the blue represents DAPI; J) Representative quantitative PCR data of genes involved in lipogenesis, FAO and OXPHOS, *n* = 3 per group; K-L) The cellular TG and TC contents in HepG2 cells with or without *H. pylori* OMVs stimulation, *n* = 3 per group; M) Representative histograms and Mean Fluorescence Intensity of ROS production assessed by flow cytometry in HepG2 cells with or without *H. pylori* OMVs stimulation; *N*) Representative confocal microscopy images with mean branch length (μm) of HepG2 cells with or without *H. pylori* OMVs stimulation stained with MitoTracker Red. Statistical analysis was performed using Two-tailed Student's *t*-test (two groups) and one-way analysis of variance (ANOVA) (multiple groups) followed by Bonferroni's test. *p < 0.05; **p < 0.01; and ***p < 0.001.

Although *H. pylori* is a gram-negative bacterium mainly colonizing the gastric mucosa, studies have demonstrated that OMVs secreted by *H. pylori* can enter the bloodstream and trigger systemic inflammatory responses, including liver injury.^15^ To characterize *H. pylori* OMVs and investigate whether *H. pylori* exerts its effects on the liver through an OMV-mediated mechanism, OMVs were first purified from the culture supernatants of the *H. pylori* 26695 strain using ultracentrifugation. The phenotypic characterization of these OMVs was assessed using TEM, immunoblotting, and NTA (Figure 2A−C), showing that OMVs derived from *H. pylori* were roughly spherical and 20 to 300 nm in size. Immunoblotting indicated that CagA, VacA, and UreaA were positive in the OMVs from the *H. pylori* 26695 strain.

We then assessed the uptake of these OMVs in vivo and in vitro. OMVs were labeled with PKH67, a membrane dye commonly used for exosomes, and subsequently co-cultured with HepG2 cells. PKH67-labeled particles were successfully internalized by HepG2 cells (Figure 2D). PKH67-labeled OMVs were gavaged into mice, and then liver immunofluorescence analysis revealed the presence of PKH67 in liver tissue (Figure 2E-F), indicating that OMVs can be taken up by the liver.

Next, we assessed the cytotoxic effects of *H. pylori* OMVs on hepatic cells using the MTT assay, which indicated that OMVs induced a slight increase in the number of viable HepG2 and L-02 cells at a concentration of 10 μg/mL, while a decrease in cell numbers was observed at 15 μg/mL (Figures 2G, S3A). Accordingly, OMVs at a concentration of 10 μg/mL were used in subsequent co-culture assays. After stimulation with free fatty acids (FFA), OMVs increased the burden of intracellular lipid droplets, as evidenced by ORO staining and immunofluorescence (Figures 2H-I, S3B-D). The mRNA expression levels of genes involved in de novo lipogenesis were significantly elevated after co-culture with OMVs, while genes associated with FAO and OXPHOS were downregulated (Figures 2J, S3E). Additionally, intracellular TC and TG levels increased significantly after OMV stimulation (Figures 2K-L, S3F-G). A significant rise in ROS levels was also observed in OMVs-treated cells (Figures 2M, S3H). Mito-tracker Red staining revealed a fragmented morphology in the mitochondria of HepG2 and L-02 cells after co-culture with OMVs (Figures 2N, S3I). These results indicate that *H. pylori* infection disrupts lipid metabolism in vitro through the mediation of OMVs.

### Identification of WTAP as a potential regulator of *H. pylori-*induced hepatic steatosis

**Figure 3.**
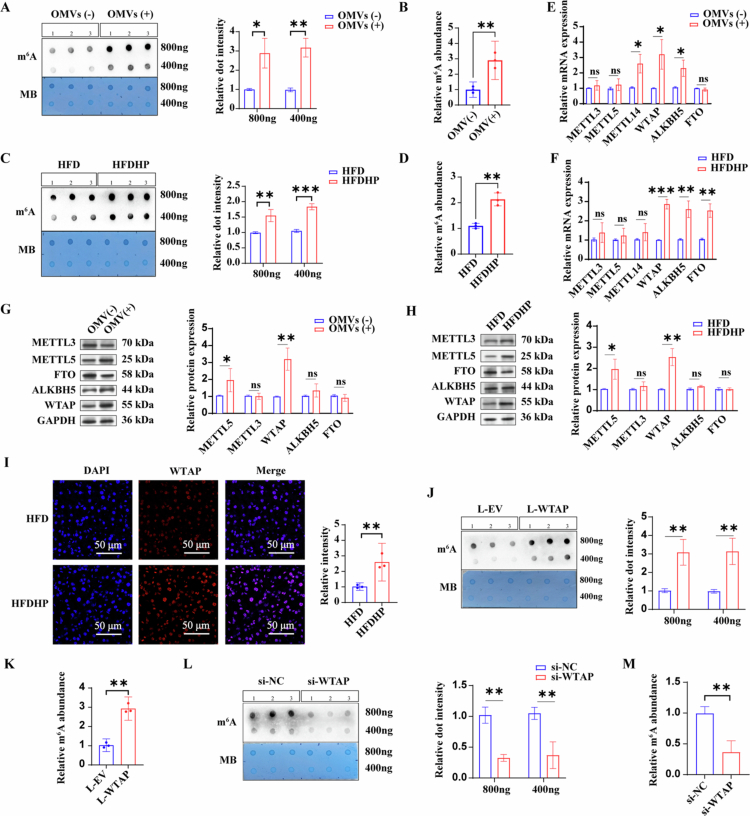
Identification of WTAP as a potential regulator of *H. pylori*-induced hepatic steatosis. A) m^6^A dot blot assay of global m^6^A abundance in HepG2 cells treated with or without *H. pylori* 26695 OMVs using 800 or 400 ng total RNAs. MB staining is used as a loading control. *n* = 3 per group; B) m^6^A RNA methylation quantification assay of global m^6^A abundance in HepG2 cells treated with or without *H. pylori* OMVs. *n* = 3 per group; C) m^6^A dot blot assay of global m^6^A abundance in liver tissues from HFD C57BL/6 mice with or without *H. pylori* infection using 800 or 400 ng total RNAs. MB staining is applied as a loading control. *n* = 3 per group; D) m^6^A RNA methylation quantification assay of global m^6^A abundance in liver tissues from HFD C57BL/6 mice with or without *H. pylori* infection. *n* = 3 per group; E-F) Relative mRNA levels of m^6^A regulators, including METTL3, METTL5, METTL14, WTAP, ALKBH5 and FTO in HepG2 cells treated with or without *H. pylori* OMVs (E) and HFD C57BL/6 mice with or without *H. pylori* infection using qPCR analysis. *n* = 3 per group; G-H) Western blotting of METTL3, METTL5, WTAP, ALKBH5 and FTO in HepG2 cells treated with or without *H. pylori* OMVs (G) and HFD C57BL/6 mice with or without *H. pylori* infection (H); GAPDH is used as an internal control. *n* = 3 per group; I) Representative images of Immunofluorescence staining of WTAP in liver tissues of HFD C57BL/6 mice with or without *H. pylori* infection; The red signals represent WTAP, while the blue represents DAPI. J) m^6^A dot blot assay of global m^6^A abundance in HepG2 transduced with L-EV or L-WTAP using 800 or 400 ng total RNAs. MB staining is applied as a loading control. *n* = 3 per group; (K) m^6^A RNA methylation quantification assay of global m^6^A abundance in HepG2 transduced with L-EV or L-WTAP; (L) m^6^A dot blot assay of global m^6^A abundance in HepG2 cells transfected with si-NC or si-WTAP using 800 or 400 ng total RNAs. MB staining is applied as a loading control. *n* = 3 per group; (M) m^6^A RNA methylation quantification assay of global m^6^A abundance in HepG2 transfected with si-NC or si-WTAP. Statistical analysis was performed using Two-tailed Student's *t*-test (two groups) and one-way analysis of variance (ANOVA) (multiple groups) followed by Bonferroni's test. *p < 0.05; **p < 0.01; and ***p < 0.001.

Previous studies have demonstrated that m^6^A modification is involved in the pathogenesis of obesity-associated fatty liver disease.^21^ To investigate whether m^6^A modification occurs in the liver after *H. pylori* infection, m^6^A levels both in vitro and in vivo were assessed. The dot blot and m^6^A RNA methylation quantification assays revealed elevated m^6^A levels in the total RNAs of HepG2 cells co-cultured with OMVs (Figure 3A-B), as well as in the livers of *H. pylori*-infected HFD mice (Figure 3C-D). To identify the specific m^6^A regulator responsible for the increased m^6^A levels after *H. pylori* infection, we assessed the expression of m^6^A writers (METTL3, METTL14, and WTAP) and erasers (ALKBH5 and FTO). The constant upregulation of WTAP mRNA and protein after *H. pylori* infection was observed both in vitro and in vivo (Figure 3E-H). RNA-seq data from livers of HFD and HFDHP mice also showed that WTAP expression was higher after *H. pylori* infection (Figure S4A). Furthermore, we also identified increased WTAP expression in liver tissues obtained from HFDHP mice through immunofluorescence staining (Figure 3I). To investigate whether WTAP directly regulates m^6^A modification, we overexpressed WTAP in HepG2 cells using a lentivirus containing the coding sequence (CDS) of the human WTAP gene, which was tagged with FLAG (L-WTAP). Immunoblotting confirmed the expression of FLAG and revealed a significant increase in WTAP expression in HepG2 cells transduced with L-WTAP (Figure S4B). We also designed three pairs of siRNAs targeting the WTAP gene (WTAP-siRNA), with WTAP-siRNA #2 selected for further assessments due to its highest efficiency as determined by qRT-PCR and western blot analysis (Figure S4C-D). The m^6^A dot blot assay indicated an increased m^6^A level in the total RNAs of HepG2 transduced with L-WTAP, while a decrease in m^6^A levels was observed in HepG2 transfected with WTAP-siRNA #2 (Figure 3J-M).

### The role of WTAP in hepatic steatosis in vivo by AAV8 tail vein injection

**Figure 4.**
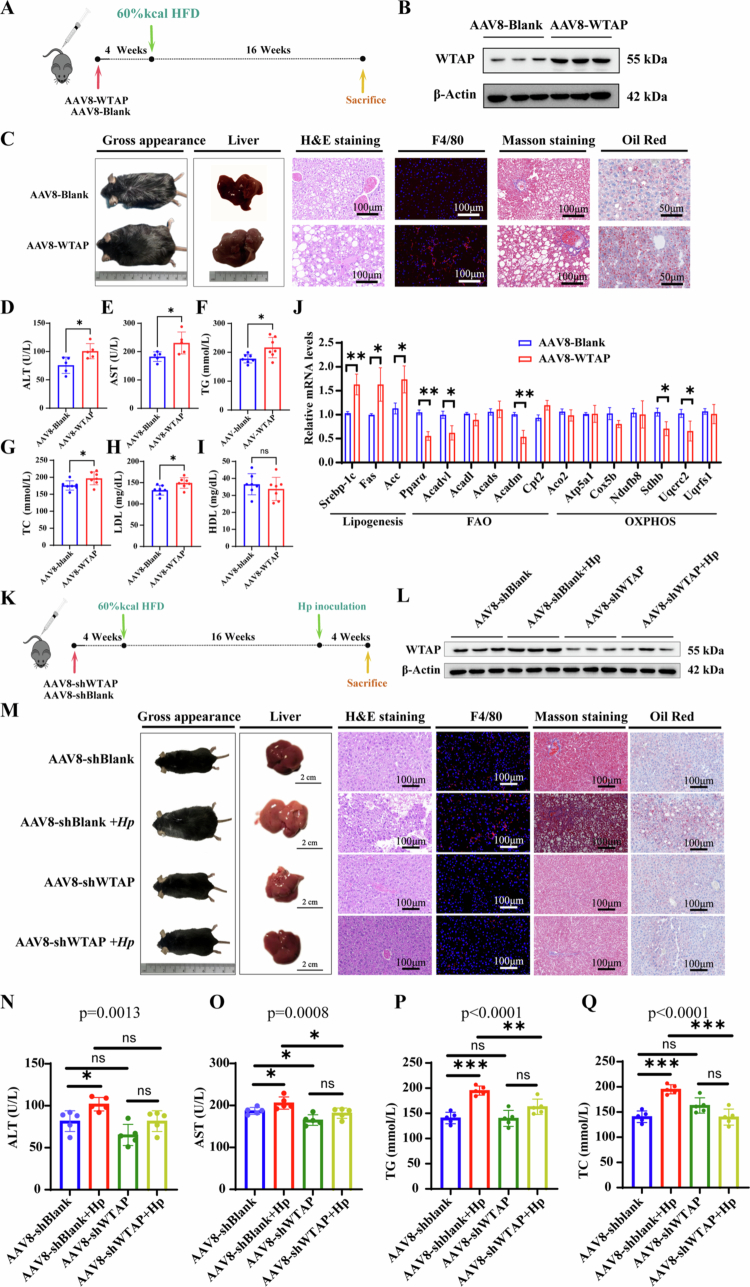
The role of WTAP in hepatic steatosis in vivo by AAV8 tail vein injection**.** A) Schematic diagram of study design using the AAV8-WTAP injection to mimic the overexpression of WTAP in C57BL/6 mice, *n* = 5 per group; B) Western blotting of WTAP expression in C57/BL6 mice treated with AAV8-WTAP or AAV8-Blank. *n* = 5 per group; C) Representative images of the gross appearance in the liver histology (1 cm), quantification of the liver index (%), H&E (100 μm), F4/80 antibody (100 μm), Masson staining (100 μm), and ORO staining (100 μm); D-I) Serum concentration of ALT (U/L) (D), AST (U/L) (E), triglyceride (mg/dL) (F), cholesterol (mg/dL) (G), LDL (mg/dL) (H), and HDL (mg/dl) (I) in C57/BL6 mice treated with AAV8-WTAP or AAV8-Blank. *n* = 5 per group; J) Representative quantitative PCR analysis with key genes involved in lipogenesis, FAO and OXPHOS metabolism in C57/BL6 mice treated with AAV8-WTAP or AAV8-Blank. *n* = 5 per group; K) Schematic diagram of study design using AAV8-shWTAP to mimic the inhibition of WTAP in C57BL/6 mice; L) Western blotting of WTAP expression in C57/BL6 mice treated with AAV8-shWTAP, AAV8-shWTAP + *H. pylori* SS1 infection, AAV8-shBlank, and AAV8-shWTAP + *H. pylori* infection. *n* = 5 per group; M) Representative images of the gross appearance in the liver histology (1 cm), quantification of the liver index (%), H&E (100 μm), F4/80 antibody (100 μm), Masson staining (100 μm), and oil red staining (100 μm). *N*-Q) Serum concentration of ALT (U/L) (*N*), AST (U/L) (O), triglyceride (mmol/L) (*P*), and cholesterol (mmol/L) (Q) in C57/BL6 mice treated with AAV8-shWTAP, AAV8-shWTAP + *H. pylori* SS1 infection, AAV8-shBlank, and AAV8-shWTAP + *H. pylori* infection. *n* = 5 per group. Statistical analysis was performed using Two-tailed Student's *t*-test (two groups) and one-way analysis of variance (ANOVA) (multiple groups) followed by Bonferroni's test. *p < 0.05; **p < 0.01; and ***p < 0.001.

To further evaluate the role of WTAP in hepatic lipid metabolism in vivo, liver specific promoter driven WTAP overexpression AAV8 (AAV8-TBG-MCS-P2A-LUC-WTAP) was employed via tail vein injection (Figure 4A), followed by in vivo imaging to confirm the efficacy of the injection (Figure S5A). Western blot confirmed the overexpression of WTAP in liver (Figure 4B). After 16 weeks of HFD feeding, mice with WTAP overexpression exhibited typical characteristics of more severe hepatic steatosis (Figure 4C), as evidenced by larger gross morphology, increased liver index, expanded ORO staining area, and elevated F4/80 signal (Figure S5B-G). We also observed that the elevation in ALT, AST, TG, TC, and LDL levels, indicating that WTAP overexpression exacerbates disturbances in lipid metabolism (Figure 4D-I). Correspondingly, hepatic WTAP overexpression significantly elevated the expression of genes associated with de novo lipogenesis, while suppressing genes related to FAO and OXPHOS (Figure 4J).

Next, we investigated the potential effects of WTAP knockdown on hepatic steatosis in vivo. WTAP knockdown was achieved through tail vein injection of AAV8-U6-MCS-CMV-fLUC-shWTAP (Figure 4K), followed by in vivo imaging to confirm injection efficacy (Figure S5H). Knockdown efficiency of WTAP expression was assessed (Figure 4L). Mice infected with AAV8-shWTAP and subjected to a 16-week HFD exhibited a remarkable reduction in body weight, decreased liver index, lighter ORO staining area, and diminished F4/80 signal compared to controls (Figure 4M). Furthermore, we observed that AAV8-shWTAP mice infected with *H. pylori* did not show more severe hepatic steatosis phenotypes, indicating AAV8-shWTAP injection mitigated the hepatic steatosis phenotype induced by *H. pylori* infection (Figures 4M, S5I-N). Additionally, we noticed notable changes to the amelioration of hepatic dysfunction with reduced serum levels of AST levels, together with TG and TC contents (Figure 4N-Q), which suggested that AAV8-mediated hepatic WTAP knockdown significantly ameliorated the fatty liver phenotype in HFD mice. Overall, these findings suggest that targeting hepatic WTAP is an effective strategy to impede the progression of liver steatosis in HFDHP mice.

### WTAP aggravates hepatic lipid metabolic dysfunction in vitro via its methylation activity

**Figure 5.**
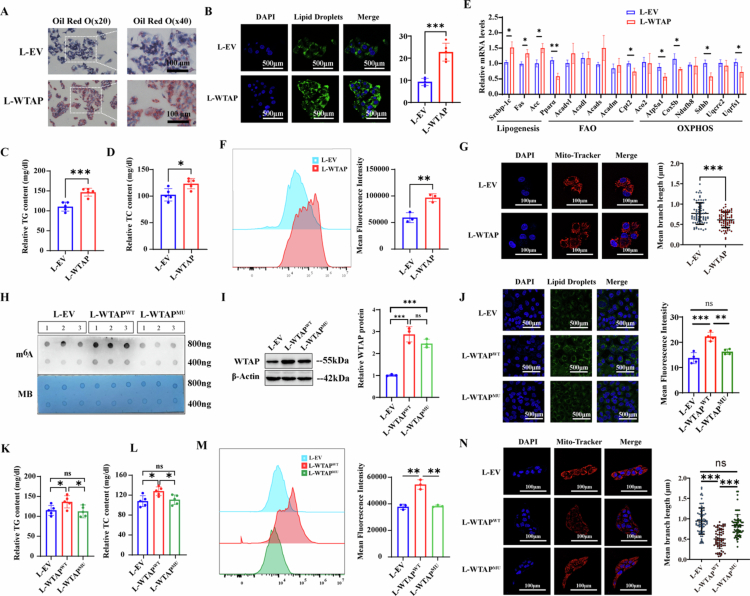
m^6^A methylation activity is necessary for WTAP to dysregulate lipid metabolism**.** A) Representative images of ORO staining of HepG2 transduced with L-EV or L-WTAP incubated with 30 μM BSA-conjugated-PA for 24 hours; B) Representative fluorescence microscopy images of HepG2 cells transfected with L-EV or L-WTAP with LDs visualized using BODIPY 493/503 staining; the green signals represent lipid droplets, while the blue represents DAPI; C-D) Concentration of Intracellular TG (C) or TC (D) in HepG2 cells transfected with L-EV or L-WTAP, *n* = 3 per group; E) Relative mRNA levels of genes involved in lipogenesis, FAO and OXPHOS in HepG2 cells transfected with L-EV or L-WTAP using qPCR analysis, *n* = 3 per group. F) Intracellular ROS levels of HepG2 cells transfected with L-EV or L-WTAP by flow cytometry, *n* = 3 per group; G) Representative confocal images of Mitochondrial Tracker Red in HepG2 cells transfected with L-EV or L-WTAP; H) m^6^A dot blot assay of global m^6^A abundance in HepG2 transduced with L-EV, L-WTAP^WT^ or L-WTAP^MU^ using 800 or 400 ng total RNAs. MB staining is used as a loading control. *n* = 3 per group. I) Immunoblotting of WTAP in HepG2 transfected with L-EV, L-WTAP^WT^ or L-WTAP^MU^. *β*-actin is used as an internal control. *n* = 3 per group; J) Representative fluorescence microscopy images of HepG2 cells transfected with L-EV, L-WTAP^WT^ or L-WTAP^MU^ with LDs visualized using BODIPY 493/503 staining; K-L) Concentration of Intracellular TG (K) or TC (L) in HepG2 cells transfected with L-EV, L-WTAP^WT^ or L-WTAP^MU^, *n* = 3 per group; M) Intracellular ROS levels of HepG2 cells transfected with L-EV, L-WTAP^WT^ or L-WTAP^MU^ by flow cytometry, *n* = 3 per group; *N*) Representative confocal images of Mitochondrial Tracker Red in HepG2 cells transfected with L-EV, L-WTAP^WT^ or L-WTAP^MU^. Statistical analysis was performed using Two-tailed Student's *t*-test (two groups) and one-way analysis of variance (ANOVA) (multiple groups) followed by Bonferroni's test. *p < 0.05; **p < 0.01; and ***p < 0.001.

To further investigate the role of WTAP in exacerbating hepatic lipid metabolism disorders in vitro, cells stably transfected with L-WTAP or L-EV were treated with 1 mM FFA for 24 hours, a concentration commonly employed as an in vitro model of hepatic steatosis. As anticipated, ORO staining (Figures 5A, S6A) and Bodipy 493/503 staining (Figures 5B, S6B) revealed a significant increase in lipid deposition in L-WTAP transfected cells. Moreover, the levels of intracellular TG (Figures 5C, S6C) and TC (Figures 5D, S6D) were significantly elevated in L-WTAP. The expression levels of key molecules involved in hepatic de novo lipogenesis were markedly increased in L-WTAP, while hepatic FAO and OXPHOS were significantly reduced (Figures 5E, S6E). Additionally, L-WTAP significantly enhanced intracellular ROS generation (Figures 5F, S6F) and induced significant mitochondrial damage (Figures 5G, S6G). These results demonstrated the role of WTAP in the reprogramming of lipid metabolism.

To further investigate whether the effects of WTAP are dependent on its methylation activity, we introduced a catalytically inactive mutation to create L-WTAP^MU^. A dot blot assay indicated that this mutation significantly inhibited the methylation activity of WTAP (Figure 5H), while immunoblotting revealed that the expression of the WTAP protein was not significantly affected (Figure 5I). The increased lipid deposition (Figure 5J), as well as the intracellular levels of TG (Figure 5K) and TC (Figure 5L), were abolished in cells transfected with L-WTAP^MU^ compared to those transfected with L-WTAP^WT^. Additionally, the overproduction of ROS (Figure 5M) and hepatic mitochondrial damage (Figure 5N) were also reduced in L-WTAP^MU^, supporting that mutation in m^6^A methylation activity reverses the promotive role of WTAP in lipid metabolic reprogramming.

### *H. pylori* infection promotes glycolysis and regulates WTAP expression via histone lactylation

**Figure 6.**
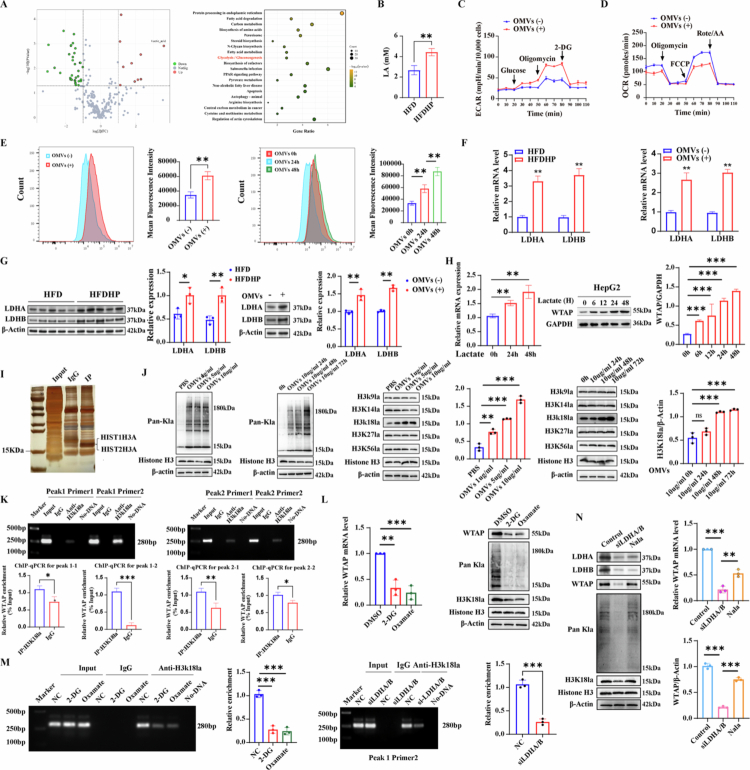
*H. pylori* infection promotes glycolysis and regulates*WTAP* expression via histone lactylation**.** A) Volcano plot of significantly-changed metabolites and KEGG analysis of remarkably changed pathways from untargeted metabolomics analysis; B) Lactic acid level of liver tissues in C57BL/6 mice with or without *H. pylori* SS1 infection, *n* = 5 per group; C) The glycolytic capability of HepG2 cells co-cultured with or without *H. pylori* OMVs using ECAR curve, *n* = 3 per group; D) Mitochondrial oxidative capacity was measured after HepG2 cells co-cultured with or without *H. pylori* OMVs using OCR curve, *n* = 3 per group; E) Intracellular ROS levels of HepG2 cells co-cultured with or without *H. pylori* 26695 OMVs in a dose or time-dependent manner, *n* = 3 per group; F-G) mRNA and protein expression of LDHA and LDHB in liver tissues or HepG2 cells following *H. pylori* 26695 infection using qRT-PCR (F) and Western blot (G) analysis, *n* = 3 per group. H) mRNA and protein expression of WTAP in HepG2 cells after lactate treatment, *n* = 3 per group; I) Representative images of Silver staining-MS of lactylated proteins; J) Pan-lysine lactylation levels in cells were measured at indicated times with 10 ug/ml *H. pylori* 26695 OMVs or after the indicated dose treatment for 24 h and lysine lactylation levels of H3 were detected under the same conditions. K) ChIP-qPCR using anti-H3K18la antibodies validates H3K18la enrichment in peak 1 and peak 2 within the promoter region of WTAP in HepG2. L) WTAP mRNA and protein level after 2-DG and oxamate treatment, *n* = 3 per group. M) ChIP-qPCR using anti-H3K18la antibodies validates H3K18la enrichment in HepG2 cells after 2-DG or oxamate treatment or si-LDHA/B transfection. *N*) WTAP mRNA and protein level in HepG2 cells after si-LDHA/B transfection with or without Nala addition, *n* = 3 per group. Statistical analysis was performed using Two-tailed Student's *t*-test (two groups) and one-way analysis of variance (ANOVA) (multiple groups) followed by Bonferroni's test. *p < 0.05; **p < 0.01; and ***p < 0.001.

We further examined the potential mechanisms of the increased WTAP expression upon *H. pylori* infection. Previous metabolomics studies have demonstrated that *H. pylori* infection and its subsequent eradication may selectively influence the metabolites present in gastric lesions.^17^ Notably, during our in vitro experiments, we observed that the co-culture with *H. pylori* OMVs resulted in a slight yellow coloration of the cell culture medium, suggesting an acidic extracellular environment (Figure S7A). Additionally, an increased concentration of lactate was detected in cells co-cultured with *H. pylori* OMVs, exhibiting a time- and dose-dependent relationship (Figure S7B). We hypothesized that *H. pylori* OMVs may mediate hepatic metabolic disturbances and contribute to the pH changes observed in the extracellular environment. Consequently, we conducted an untargeted metabolomics analysis, revealing significant activation of the glycolysis and gluconeogenesis pathways, along with elevated lactic acid levels in the livers of HFDHP mice (Figure 6A-B).

Lactate, a byproduct of cellular glycolysis, is the primary factor in creating an acidic microenvironment within cells. To investigate whether *H. pylori* OMVs modify the glycolysis process in hepatic cells, we measured the extracellular acidification rate to assess glycolysis levels. Results demonstrated that *H. pylori* infection considerably increased cellular glycolysis, impacting both the basal and maximal phases (Figure 6C). OCR was reduced after *H. pylori* infection, suggesting inhibition of aerobic respiration (Figure 6D). Using glucose uptake assay, we further demonstrated increased glucose uptake, which was supported by the increased intracellular fluorescent intensity of NBD-glucose after *H. pylori* OMVs co-culture (Figure 6E). Meanwhile, we observed LDHA and LDHB, as two major glycolysis-related genes, were also increased significantly after *H. pylori* infection in vitro and in vivo (Figure 6F-G). The GEO public dataset (GSE260666) also suggested that WTAP expression was higher in livers of MASH patients and WTAP expression is positively associated with lactate dehydrogenase LDHA (Rho 0.77, *p* = 0.016) and LDHB (Rho 0.80, *p* = 0.010) expression (Figure S7C). These results indicated that *H. pylori* infection promotes hepatic glycolysis and therefore increases lactate accumulation.

Next, we aimed to further ascertain whether lactate is responsible for the WTAP up-regulation. Adding lactate into the culture medium of HepG2 cells resulted in a time-dependent increase in both mRNA and protein expression of WTAP (Figure 6H), suggesting that lactate may be a contributing factor to the up-regulation of WTAP. We further investigated whether histone lactylation could serve as a mechanism facilitating the expression of WTAP. After adding *H. pylori* OMVs to HepG2 cells, we observed elevated levels of Pan-Kla, H3K18la, and WTAP expression, as demonstrated by western blotting and immunofluorescence (Figures 6I-J and S7D). The ChIP-Seq analysis (GEO accession number: GSE156675) with anti-H3K18la antibodies identified substantial enrichment of the H3K18la signal, highlighted by two peaks in the promoter region of the WTAP gene (Figure S7E). Further validation by ChIP-qPCR showed that H3K18la was abundant in peak 1 (Figure 6K). To attenuate lactate production and histone lactylation, we employed glycolysis inhibitors, 2-DG and oxamate, as well as siRNAs targeting LDHA and LDHB, as previously reported (Figures S7F, 6L). The ChIP-qPCR assay confirmed a decrease in H3K18la, along with reductions in WTAP expression, global lactylation, and H3K18la levels in HepG2 cells (Figure 6M). Furthermore, the addition of sodium lactate (Nala) to LDHA/LDHB-deficient cells partially restored levels of WTAP and histone lactylation (Figure 6N). Upon inhibiting glycolysis through the application of 2-DG and oxamate, *H. pylori* OMVs no longer exacerbated steatosis in HepG2 cells (Figure S8A).

### Lactylation of WTAP enhances the capture of RNA that is modified with m^6^A

**Figure 7.**
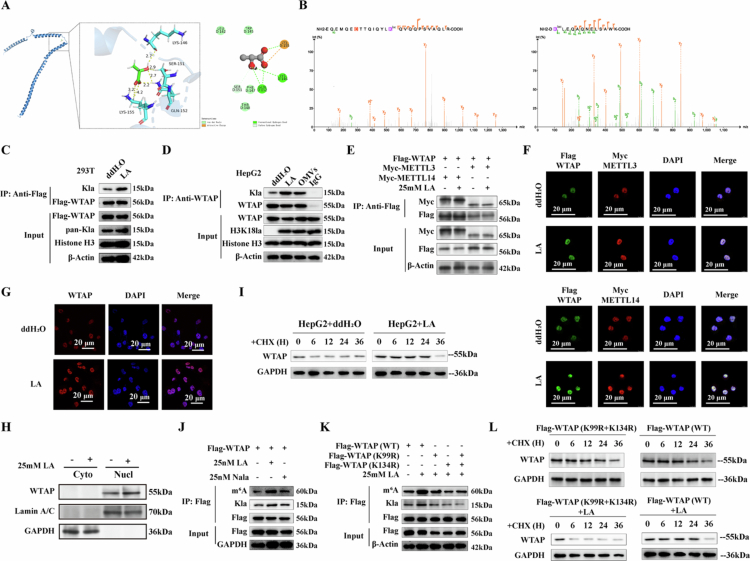
Direct lactylation of WTAP enhanced the capture of m6A-modified RNA. A) MOE is used to predict the binding affinity between the WTAP protein and L-lactate; B) Illustration of WTAP Kla sites derived from HepG2 cells identified by LC-MS; C) Lactylation of WTAP in 293 T cells was confirmed by IP method. 293 T cells transfected with FLAG-WTAP, treated with ddH2O or 25 mM L-lactic acid for 24 h. The cells were lysed for SDS pre-treated immunoprecipitation with anti-FLAG antibody followed by western blotting; D) Lactylation of WTAP in HepG2 was confirmed by IP method. HepG2 co-cultured with OMVs or treated with 25 mM LA for 24 h. The cells were lysed for SDS pre-treated immunoprecipitation with anti-METTL3 antibody followed by western blotting; E) FLAG-WTAP, with Myc-METTL3 or Myc-METTL14 were transfected into 293 T cells for 24 h. Then cells were treated with 25 mM L-lactic acid for 24 h. Lysates were used for IP with anti-FLAG antibody, followed by western blotting; F) FLAG-WTAP, with Myc-METTL3 or Myc-METTL14 were transfected into 293 T cells for 24 h. Then cells were treated with 25 mM L-lactic acid for 24 h. Cells were harvested for immunofluorescence staining and further photographed by laser scanning confocal microscopy. Scale bars, 20 mm; G) HepG2 were treated with 25 mM L-lactic acid for 24 h. Cells were harvested for immunofluorescence staining (20 mm) and further photographed by laser scanning confocal microscopy; H) Western blotting analysis of the distribution in nuclear and cytoplasmic fractions of WTAP in HepG2 treated with or without 25 mM L-lactic acid for 24 h. I) Western blotting analysis of WTAP in HepG2 with or without lactic acid treated with 0.1 mg/mL CHX for indicated time; J) FLAG-WTAP was transfected into 293 T cells for 24 h. Then cells were treated with L-lactic acid or Nala for 24 h. Cells were 254 nm UV-crosslinked before harvesting. Lysates were used for SDS pre-treated immunoprecipitation with anti-FLAG antibody, followed by western blotting to detect indicated targets. K) FLAG-WTAP (WT), FLAG-WTAP (K99R), FLAG-WTAP (K134R), and FLAG-WTAP (K99R and K134R) were transfected into 293 T cells for 24 h. Then cells were treated with L-lactic acid for 24 h. Cells were 254 nm UV-crosslinked before harvesting. Lysates were used for SDS pre-treated immunoprecipitation with anti-FLAG antibody, followed by western blotting to detect indicated targets; L) Western blotting analysis of WTAP in 293 T cells transfected with FLAG-WTAP (WT) and FLAG-WTAP (K99R and K134R) with or without lactic acid treated with 0.1 mg/mL CHX for indicated time.

Post-translational modifications (PTMs) with significant functions have been identified in m^6^A writers, notably lactylation driven by METTL3, which has been reported to enhance its methyltransferase activity.^24^ Metabolites can directly induce specific PTMs of proteins.^25^ We are also interested in evaluating the direct modification pattern of lactate to modulate WTAP's function. To estimate the binding affinity of L-lactic acid molecules with WTAP, molecular docking was conducted in the molecular operating environment (MOE). Several amino acid sites were predicted to be lactylated (Figure 7A). Furthermore, LC-MS/MS was utilized to verify specific lactylation modification sites within the WTAP protein. The findings identified two potential lactylation sites, K99 and K134 (Figure 7B). The two lysine residues across different species were highly conserved (Figure S8B). To determine whether WTAP can be lactylated in cells, we transiently transfected FLAG-WTAP into HEK-293T cells and added 25 mM lactic acid to the culture medium. To eliminate indirect modifications, we disrupted protein-protein interactions using sodium dodecyl sulfate (SDS) before performing anti-FLAG immunoprecipitation. Western blotting confirmed the presence of lactylation on WTAP (Figure 7C). Importantly, WTAP lactylation was also detected in HepG2 cells after co-culturing with *H. pylori* OMVs for 48 hours (Figure 7D).

RNA m^6^A methylation is predominantly facilitated by an MTase complex composed of WTAP, METTL3, and METTL14.^26^ Our investigation focused on whether lactylation of WTAP modifies its interaction with METTL3 and METTL14. Endogenous IP was conducted with an anti-WTAP antibody, and the results from immunoblotting analysis indicated that the lactylation of METTL3 did not alter its binding with METTL14 and WTAP (Figure 7E), consistent with the findings from immunofluorescence staining (Figure 7F). To assess the impact of lactylation on the nuclear localization of WTAP, FLAG-WTAP transfected 293 T cells were subjected to extraction for the separation of cytoplasmic and nuclear protein fractions. The findings indicated that there was no alteration in localization following treatment with lactic acid (Figure 7G-H). Then we analyzed the stability of WTAP in the presence or absence of lactic acid for 36 h, followed by cyclohexane (CHX) to inhibit cellular protein generation. The increased half-life of WTAP indicated that lactylation enhanced its stability (Figure 7I).

Additionally, we explored the impact of METTL3 lactylation on its interaction with m^6^A-modified RNA. The following variants: FLAG-WTAP, FLAG-WTAP (K99R), FLAG-WTAP (K134R), and FLAG-WTAP (K99R, K134R) were introduced into 293 T cells through transfection. After UV crosslinking, immunoprecipitation was carried out on the lysed cells with an anti-FLAG antibody, and western blotting was then conducted using an anti-m^6^A antibody. The results indicated that the m^6^A-modified RNAs bound by FLAG-WTAP-Kla were more abundant than those associated with FLAG-WTAP (Figure 7J). However, the level of m^6^A-modified RNAs bound by FLAG-WTAP-K99R, -K134R, or double mutants, was much less than those bound by FLAG-WTAP-WT (Figure 7K). Additionally, FLAG-WTAP-K99R, -K134R, or double mutants failed to increase lipid droplets compared with FLAG-WTAP-WT (Figure S8C). WTAP mutants (K99R, K134R) transfection results in a reduced half-life of WTAP protein after lactic acid treatment, while no change in WTAP protein stability without lactic acid exposure (Figure 7L).

### WTAP regulates GLUT3 mRNA stability with YTHDF1 as the reader in an m^6^A-dependent manner

**Figure 8.**
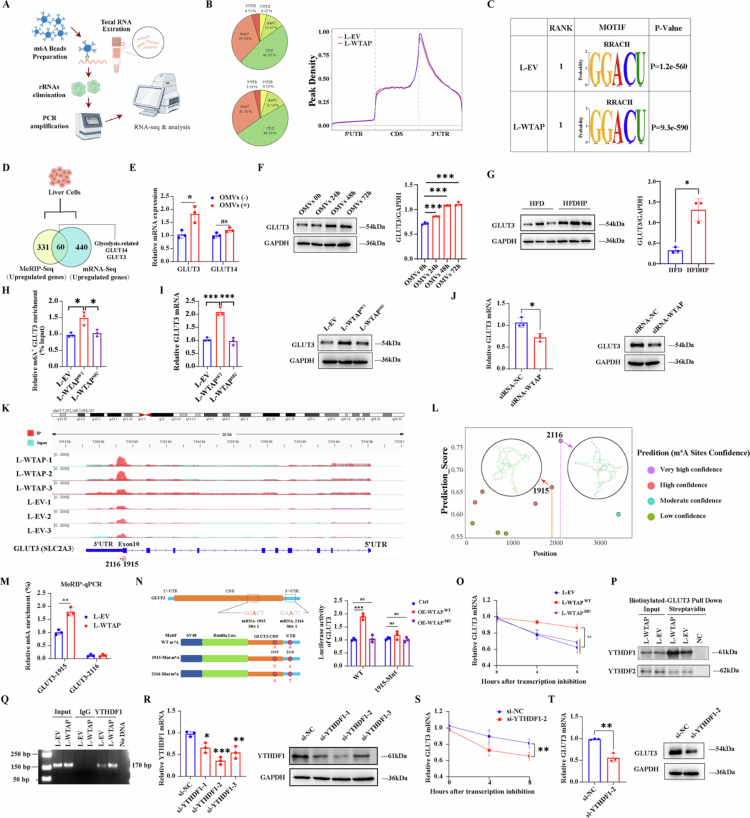
WTAP regulates GLUT3 mRNA stability with YTHDF1 as the reader in an m6A-dependent manner**.** A) Workflow for methylated RNA immunoprecipitation sequencing (MeRIP-seq); B) The distribution and Peak Density of total m^6^A peaks in distinct regions of mRNA transcripts: (Left) Pi chart showing the proportional distribution of all identified m^6^A peaks across five distinct regions of mRNA transcripts: 5'untranslated region (5'UTR), coding sequence (CDS), 3' untranslated region (3'UTR), start codon (Start C), and stop codon (Stop C). (Right) Metagene profile showing the density of m^6^A peaks across a normalized transcript model. The plot illustrates peak density throughout the 5'UTR (including the start codon region), CDS, and 3'UTR (including the stop codon region). Lines represent data from L-EV (red) and L-WTAP group (blue); C) Sequence of enriched motif displayed by HOMER; D) Venn plot of genes containing m6A-hyper peaks with altered mRNA expression which were sorted out and the gene list showed the glycolysis-related genes in the intersection; E) Relative mRNA levels of GLUT3 and GLUT14 in HepG2 cells with or without *H. pylori* 26695 OMVs co-culture, *n* = 3 per group; F) The protein level of GLUT3 in HepG2 cells with or without *H. pylori* OMVs co-culture for indicated time, *n* = 3 per group; G) The protein level of GLUT3 in C57BL/6 mice with or without *H. pylori* infection, *n* = 3 per group; H) MeRIP-qPCR analysis of m^6^A enrichment on GLUT3 mRNA in HepG2 cells transduced with L-EV, L-WTAP^WT^ or L-WTAP^MU^, *n* = 3 per group; I) mRNA and protein expression of GLUT3 in HepG2 transduced with indicated lentivirus. GAPDH is used as an internal control. *n* = 3 per group; J) GLUT3 mRNA and protein levels in HepG2 cells transfected with scramble siRNA or WTAP-siRNA. GAPDH was used as an internal control. *n* = 3 per group; K) IGV plots of m^6^A peaks in m^6^A immunoprecipitation sample (relative to input sample) at GLUT3 mRNA in HepG2 stably transfected with L-WTAP cells related to L-EV. The y-axis shows normalized reads coverage; L) Prediction of m^6^A modification sites. The secondary structure of highconfidence prediction sites is shown; M) MeRIP-qPCR assay validating that GLUT3-1915 shows significant methylation activity compared to GLUT3-2116, using L-EV and L-WTAP cell lines (*n* = 3); *N*) The structure chart showing the predicted m^6^A motifs in the CDS and 3'UTR of GLUT3 according to the result of MeRIP-seq. Relative luciferase activity of WT or Mut (A-to-T mutation) of GLUT3 luciferase reporter in Ctrl, WTAP^WT^-OE and WTAP^MU^-OE cell lines; O) qRT-PCR analysis of GLUT3 mRNA decay rate at the indicated times after actinomycin D (2 μg/ml) treatment in HepG2 cells transduced with L-EV, L-WTAP^WT^ or L-WTAP^MU^ with expression normalized to that of *β*-actin (*n* = 3); *P*) Western blotting analysis of YTHDF1 and YTHDF2 after RNA pull-down assay with biotinylated-Glut3; Q) RNA immunoprecipitation confirmed the direct binding between the YTHDF1 protein and the GLUT3 transcript, *n* = 3 per group; R) YTHDF1 mRNA levels and protein expression detected by qPCR and western blotting in HepG2 cells transfected with distinct siRNAs, *n* = 3 per group; S) qRT-PCR analysis of GLUT3 mRNA decay rate at the indicated times after actinomycin D (2 μg/ml) treatment in HepG2 cells transfected with si-NC or si-YTHDF1; T) CDK2 mRNA and protein levels in HepG2 cells transfected with scramble siRNA or YTHDF1-siRNA. GAPDH is used as an internal control. Statistical analysis was performed using Two-tailed Student's *t*-test (two groups) and one-way analysis of variance (ANOVA) (multiple groups) followed by Bonferroni's test. *p < 0.05; **p < 0.01; and ***p < 0.001.

Since WTAP mediates hepatic lipid reprogramming through its methylation activity, we then applied methylated RNA immunoprecipitation-sequencing (MeRIP-Seq) to identify downstream targets of WTAP in HepG2 cells (Figure 8A). A total of 668 m^6^A-hyper peaks and 1388 m^6^A-hypo peaks (Log_2_FC > 0.5 or < −0.5; *p* < 0.05) were initially identified in L-WTAP compared to L-EV. MeRIP-Seq revealed a dominant distribution of m^6^A peaks located in CDS and 3'-UTR of mRNA transcripts (Figure 8B). Using the HOMER motif discovery tool, we identified that the primary motif enriched in m^6^A sites was the “GGACU” consensus sequence (Figure 8C). Given the methylation activity of WTAP, we concentrated on genes exhibiting m^6^A hypermethylation peaks that also displayed dysregulated mRNA expression, as indicated by the RNA-seq data. A total of 60 genes were identified (Figure 8D). We selected GLUT3 and GLUT14 as potential downstream targets of WTAP for further investigation due to their association with glycolysis. Elevated GLUT3 mRNA expression was observed in HepG2 cells exposed to *H. pylori* OMVs, while GLUT14 mRNA expression remained unchanged (Figure 8E). Consequently, we focused on GLUT3 as a potential key regulator of WTAP in our subsequent studies. Additionally, increased GLUT3 protein expression was detected in HepG2 cells exposed to *H. pylori* OMVs (Figure 8F) and in the livers of HFDHP mice (Figure 8G).

We next investigated whether GLUT3 was a direct target of WTAP. MeRIP-qPCR identified increased m^6^A level in the GLUT3 transcript in HepG2 overexpressing wild type WTAP, but not its mutant form (Figure 8H). According to findings from qPCR and immunoblotting, the expression levels of both GLUT3 mRNA and protein were elevated in HepG2 following the transduction of L-WTAP^WT^; however, this effect was negated in L-WTAP^MU^ (Figure 8I). Consistently, the levels of GLUT3 mRNA and protein were diminished in HepG2 cells that were transfected with WTAP-siRNA (Figure 8J). Overall, our findings suggest that WTAP influences the expression of GLUT3 by methylating the GLUT3 transcript. Integrative genomics viewer (IGV) analysis of enriched m^6^A peaks of GLUT3 showed an increased m^6^A level in L-WTAP cells compared with L-EV cells, indicating that WTAP may promote m^6^A modification of GLUT3 mainly in the CDS and 3'UTR regions (Figure 8K). To further explore the detailed modification site, we used SRAMP, a predictor for m^6^A modification sites based on sequences. By combining the prediction outcomes with our motif, GGACU, we recognized two probable m^6^A modification locations with considerable confidence: adenine 1915 in the CDS area and adenine 2116 in the 3' untranslated region (3'UTR) of the GLUT3 transcript (Figure 8L). The subsequent MeRIP-qPCR analysis utilizing specific primers demonstrated that the 1915 site on the GLUT3 transcript serves as the direct target for methylation mediated by WTAP (Figure 8M). Using the wild type luciferase reporter, we designed the GLUT3-1915-mut luciferase reporter by changing the specific adenosine (A) in the m^6^A motif to thymine (T). The results of the dual luciferase assay indicated that WTAP^WT^-OE but not WTAP^MU^-OE could promote the luciferase activity of GLUT3^WT^ while both WTAP^WT^-OE and WTAP^MU^-OE did not boost the luciferase activity of GLUT3^MU^ (Figure 8N).

Next, we annotated the specific m^6^A reader that binds to GLUT3. Above data suggested that WTAP increased m^6^A level and mRNA expression of GLUT3, therefore YTHDF1, a well-recognized m^6^A reader that promotes targeted mRNA stability, was selected as a potential binding protein to GLUT3. Subsequently, we examined whether WTAP enhances GLUT3 expression by stabilizing its mRNA, with YTHDF1 serving as a reader. The detection of a prolonged half-life for the GLUT3 transcript in cells with L-WTAP^WT^, but not in those with the mutant WTAP protein, supports the notion that WTAP strengthens GLUT3 mRNA stability through its methylation activity (Figure 8O). RNA pull down assay and RIP further confirmed the direct binding between the YTHDF1 rather than YTHDF2 protein and the GLUT3 transcript (Figure 8P-Q). We then analyzed whether YTHDF1 knockdown regulated GLUT3 mRNA decay. Among all three pairs of siRNAs targeting the YTHDF1 gene, YTHDF1-siRNA#2 presented the highest efficiency and was chosen for the following studies (Figure 8R). Stability of the GLUT3 mRNA was decreased upon YTHDF1 knocking down (Figure 8S), followed by decreased expression of the GLUT3 mRNA and protein (Figure 8T). Previously reported m^6^A, RIP and CLIP data from the m^6^A2Target Database (http://m6a2target.canceromics.org) also identified GLUT3 mRNA as a potential target of YTHDF1 on the 3' UTR or exon sites. The GEO public dataset (GSE260666) also suggested that mRNA expression of YTHDF1 and pro-GLUT3 was higher in livers of MASH patients (Figure S8D). Furthermore, we knocked down GLUT3 and YTHDF1 by siRNAs under conditions of *H. pylori* infection (Figure S8E-G), and subsequently evaluated the phenotypes associated with MASLD. We found that a significant reduction in the accumulation of intracellular lipid droplets, as indicated by ORO staining, after inhibiting the expression of YTHDF1 (Figure S8E) or GLUT3 (Figure S8G), compared to their si-NC controls. Collectively, these findings indicated that WTAP regulates GLUT3 mRNA stability in an m^6^A-YTHDF1-dependent manner.

### *H. pylori-*induced hepatic steatosis in HFDHP mice is alleviated after*H. pylori* eradication

**Figure 9.**
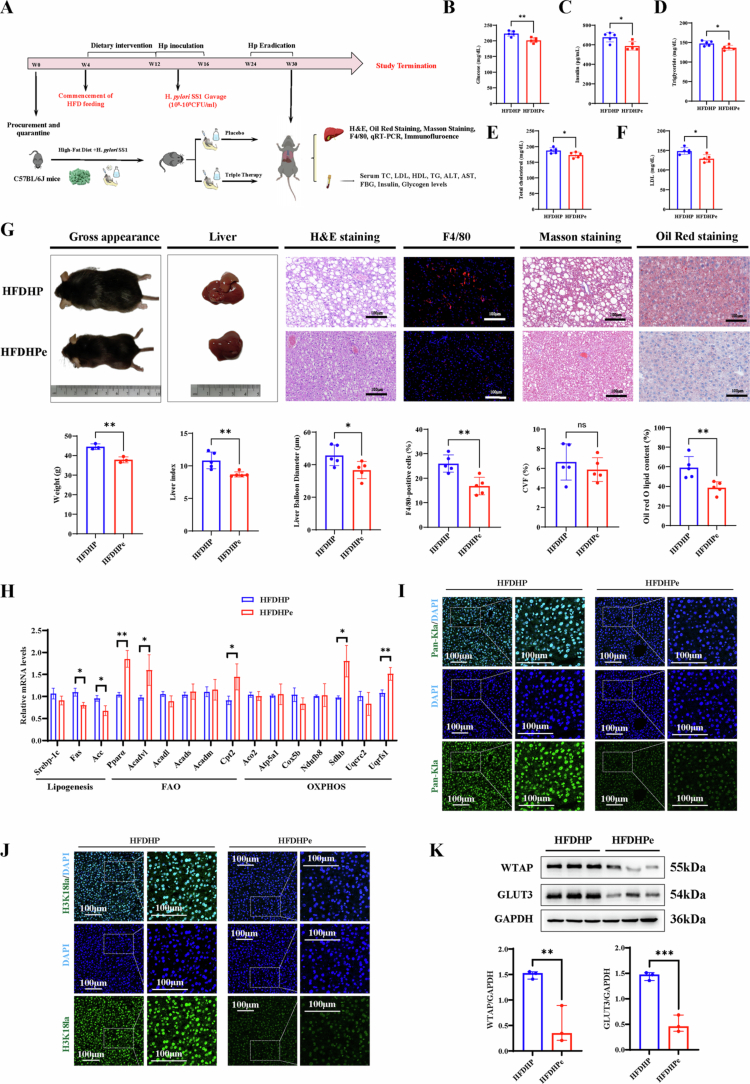
*H. pylori-*induced hepatic steatosis in HFDHP mice is alleviated after*H. pylori* eradication. A) Schematic diagram of the animal experiment; C57BL/6 mice with HFD diets were divided into HFDHP (*H. pylori SS1* infection) and HFDHPe (*H. pylori* eradication) groups; *n* = 5 per group; B−F) Serum concentration of glucose (mg/dL) (B), insulin (pg/mL) (C), triglyceride (mg/dL) (D), cholesterol (mg/dL) (E), and LDL (mg/dL) (F) in *H. pylori*-infected C57/BL6 mice eradicated with triple therapy or placebo. *n* = 5 per group; G) Representative images of the gross appearance in the liver histology (1 cm), quantification of the liver index (%), H&E (100μm), F4/80 antibody (100μm), Masson staining (100μm), and ORO staining (100μm). Histogram plots of weight (g), liver index (%), average liver balloon diameters (μm), number of F4/80 positive cells (%), CVF (%), and oil red lipid contents (%) of *H. pylori*-infected C57/BL6 mice eradicated with triple therapy or placebo. *n* = 5 per group; H) Representative quantitative PCR analysis with key genes involved in lipogenesis, FAO and OXPHOS metabolism in *H. pylori*-infected C57/BL6 mice eradicated with triple therapy or placebo. *n* = 5 per group; I−J) Immunofluorescence staining of Pan-Kla (I) and H3k18la (J) expression in *H. pylori*-infected C57/BL6 mice eradicated with triple therapy or placebo. *n* = 5 per group; K) Western blotting of WTAP and GLUT3 expression in *H. pylori*-infected C57/BL6 mice eradicated with triple therapy or placebo. *n* = 5 per group. Statistical analysis was performed using Two-tailed Student's *t*-test. *p < 0.05; **p < 0.01; and ***p < 0.001.

**Figure 10.**
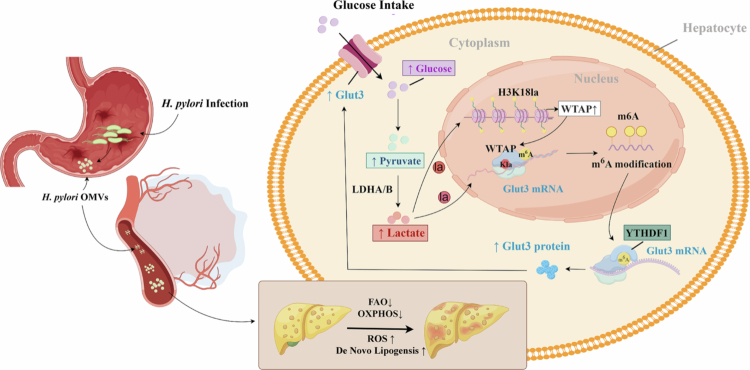
Schematic diagram of WTAP-mediated effects on *H. pylori* infection-induced lipid metabolic disorders**.** The up-regulation of WTAP in the liver, driven by histone and direct lactylation due to *H. pylori* infection, increases lipogenesis while decreasing FAO and OXPHOS, thereby exacerbating lipid metabolic dysfunction. WTAP mediates liver steatosis phenotypes by regulating the mRNA stability of GLUT3, with YTHDF1 acting as the reader in an m6A-dependent manner.

To investigate the impact of *H. pylori* eradication on liver lipid metabolic reprogramming, *H. pylori*-infected mice were subjected to a triple therapy regimen comprising clarithromycin, rabeprazole, and amoxicillin (Figure 9A). FBG, LDL, TG, and total TC levels were found to be lower in the eradicated mice compared to those receiving a placebo (Figure 9B-F). ALT, AST, HDL and glycogen levels did not show significant differences between the two groups (Figure S9A-D). The impaired glucose intolerance and insulin sensitivity were alleviated after eradication (Figure S9E-F). Furthermore, liver microvesicular steatosis and the presence of infiltrating macrophages were also diminished in the eradicated mice, indicating an improvement in hepatic lipid dysfunction following *H. pylori* eradication (Figure 9G). qRT-PCR analysis suggested that *H. pylori* eradication effectively increased the expression of genes involved in FAO and OXPHOS, while significantly suppressing the genes associated with de novo lipogenesis (Figure 9H). Immunoblotting and immunofluorescence analyzes indicated reduced expression levels of WTAP, GLUT3, Pan-Kla, and H3K18la in the eradicated mice (Figure 9I-K). Collectively, these data suggest that the WTAP/GLUT3 axis plays a role in promoting *H. pylori*-induced liver steatosis, partly through the activation of glycolysis (Figure 10).

## Discussion

Accumulating evidence indicates that *H. pylori* infection is associated with an elevated risk of both prevalent and incident MASLD,^10-13^ although the underlying regulatory mechanisms have yet to be fully elucidated. Our observations revealed disruptions in lipid metabolism in both in vivo and in vitro models, accompanied by a significant increase in hepatic m^6^A levels following *H. pylori* infection. Recent studies have highlighted the crucial role of m^6^A RNA modification in metabolic disorders,^18^^,^^27^ and it has been shown that *H. pylori* infection affects m^6^A methylation levels and patterns in the progression of gastric cancer and the regulation of gene expression.^28-30^ However, to date, no studies have investigated m^6^A RNA modification in liver steatosis induced by *H. pylori* infection. Upon screening m^6^A methyltransferases, we found that only WTAP was consistently upregulated in the liver. Consequently, we focused on the critical role of WTAP as a regulatory subunit of the m^6^A methyltransferase complex.^31^ WTAP facilitates the recruitment of the METTL3-METTL14 heterodimer to nuclear speckles and is essential for the complex's full enzymatic functionality. Previous research has indicated that the knockdown of WTAP alone leads to a significant reduction in global m^6^A levels, while METTL3 and METTL14 maintain normal expression levels.^32^^,^^33^ This suggests that WTAP serves as a limiting factor for the overall function of the m^6^A methylation complex. While lactylation of the catalytic subunit METTL3 has been reported to enhance m^6^A deposition in cancer immunity,^24^ our study unveils a distinct regulatory mechanism centered on the regulatory subunit WTAP in the context of infection-driven MASLD. Importantly, the lactylation of WTAP enhances its RNA-binding capacity without disrupting its partnership with METTL3/METTL14, representing a complementary layer of regulation within the m^6^A methyltransferase complex. We postulate that this enhanced binding to m6A-modified transcripts establishes a positive feedback loop, potentially protecting nascent m^6^A marks from immediate demethylation and facilitating reader protein recruitment, thereby leading to the net accumulation of m^6^A observed in our study. Notably, a prior study reported that hepatic knockout of WTAP exacerbates diet-induced NASH, a finding that seems to contrast with our results.^34^ This apparent discrepancy likely highlights the context-dependent functionality of the m^6^A machinery. The study by Li et al. utilized a purely metabolic model, where global WTAP deletion may induce compensatory systemic responses, such as increased adipose tissue lipolysis. In contrast, our model focuses on pathogen infection, specifically examining how *H. pylori* modulates WTAP activity through lactylation, thereby activating a distinct cell-autonomous pathway via the YTHDF1-GLUT3 axis. This comparison suggests that the role of WTAP is not uniform but is influenced by the underlying disease trigger, underscoring the novelty of our findings in the context of infection-associated MASLD.

While primarily colonizing the stomach, *H. pylori* has also been implicated in the progression of various extragastrointestinal conditions.^7^ There is limited knowledge about how *H. pylori* causes these diseases beyond the gastrointestinal system. Systemic inflammation and immune dysregulation were suggested to contribute to metabolic diseases progression, including diabetes and MASLD, after *H. pylori* infection.^11^^,^^12^^,^^35^ Our study suggested that the bacteria derived OMVs may be the mediators responsible for disrupting hepatic lipid metabolism. Upon direct gavage of OMVs into the stomach, although differing from the continuous, lower-level secretion that occurs in the context of a natural infection, these vesicles were subsequently detected in the liver tissue of the mice, demonstrating the capability of OMVs to survive the harsh gastrointestinal environment, cross the gut epithelial barrier, and subsequently be taken up by hepatocytes. CagA and VacA,^36^^,^^37^ recognized as the principal virulence factors in the pathogenesis of *H. pylori*-related diseases, were reported to be packaged into OMVs to interact with epithelial cells, influencing host gene transcription and potentially leading to severe clinical outcomes. Interestingly, we found that *H. pylori* infection alone, in the absence of a high-fat diet, does not significantly induce hepatic steatosis or inflammation. This observation aligns with the complex, multifactorial nature of MASLD pathogenesis. The “two-hit hypothesis,” as proposed by Day et al.^38^ is a widely accepted framework in the progression of MASLD. According to this hypothesis, the “first hit”-such as insulin resistance, genetic predisposition, or, in our study, high-fat diets-sensitizes the liver to subsequent damage. The “second hit,” which may involve factors such as *H. pylori* infection, WTAP upregulation, oxidative stress, and inflammatory cytokines, is necessary to precipitate the clinical manifestations of steatosis, inflammation, and fibrosis. We also found in our mouse model that the unchanged CVF and *α*-SMA staining of the liver after *H. pylori* infection, which might be due to the acute nature of the hepatic inflammation, where hepatic stellate cells (HSC) activation may not yet be evident, or because collagen deposition is regulated by other mechanisms independent of HSCs. Alternative fibrotic pathways, such as through RNA-seq or other HSC activation markers (e.g., PDGFR-*β*), will be investigated in future studies.

We also identified *H. pylori* infection as a novel upstream pathogenic trigger that activates WTAP through lactylation, a newly found post-translational modification. This hypothesis that lactylation modification may occur upstream of WTAP is based on our initial observation that co-culturing liver cells with *H. pylori* OMVs led to a subtle yellow discoloration of the hepatocytes culture medium, suggesting the presence of an acidic extracellular environment and an increase in glycolytic activity, as evidenced by ECAR and OCR. Lactylation significantly impacts various diseases by affecting protein interactions, gene expression, and immune responses.^39^ Lactate-induced lactylation at histone H3K18 can influence downstream m^6^A modifications by boosting the transcription levels of enzymes that regulate m^6^A.^40^^,^^41^ *H. pylori*-induced lactate operates through a dual mechanism involving both transcriptional and post-translational modifications: 1) at the transcriptional level, lactate-mediated H3K18la associates with the WTAP promoter, thereby modulating WTAP mRNA expression; 2) at the post-translational level, lactate directly lactylates the WTAP protein, thereby enhancing its stability and function. Any disruption in this lactate-lactylation pathway-whether through a reduction in lactate levels or mutations at the lactylation sites-significantly impairs the *H. pylori*-mediated enhancement of WTAP function, affecting both its expression and activity. Consequently, targeting lactylation may present therapeutic potential for MASLD and it is important to recognize that additional factors may also play a role in the up-regulation of WTAP following *H. pylori* infection, considering the complex etiology involved. Furthermore, we delineate the downstream axis (YTHDF1-GLUT3) and provide genetic evidence that this axis is indispensable for *H. pylori*-mediated MASLD pathogenesis. Although the role of WTAP in hepatic lipid metabolism has been previously investigated,^34^ the downstream effector of WTAP remains unexplored. By employing RNA-seq and MeRIP-seq, we identified that GLUT3 (SLC2A3) and GLUT14 (SLC2A14) were the ones directly associated with glucose metabolism and glycolysis. Further validation showed that *H. pylori* infection and OMVs significantly upregulated GLUT3 mRNA and protein levels, while GLUT14 expression remained unchanged, indicating GLUT3 is a compelling downstream target of WTAP. GLUT3 predominantly facilitates the neuronal transport of glucose; however, it is also expressed at lower levels in various other organs, including the liver.^42^^,^^43^ As a glucose transporter, GLUT3 is essential for mediating glucose uptake and is intricately associated with the glycolytic pathway.^43^ The overexpression of GLUT3 was found to enhance tumor progression and metastasis,^44^ underscoring its potential significance in therapeutic interventions. Our results showed that the upregulation of WTAP expression enhances the m^6^A modification of GLUT3, leading to increased levels of hepatic GLUT3 protein. GLUT3 upregulation enhances glycolysis, leading to lactate production. This lactate, in turn, fuels a positive feedback loop by promoting WTAP expression (via H3K18la) and function (via direct lactylation). This established a coherent pathogenic circuit directly linking *H. pylori* infection to enhanced glycolytic flux and lipid accumulation via the WTAP-YTHDF1-GLUT3 axis. Future studies are warranted to confirm the role of GLUT3 in vivo in *H. pylori*-induced MASLD using inducible hepatocyte-specific GLUT3 knockout models.

Our study has several limitations. Firstly, we employed solely the AAV8 adenovirus to disrupt WTAP expression, rather than utilizing the knockout mouse model. Secondly, we conducted Western blot analysis on key components of *H. pylori-*derived OMVs without undertaking specific proteomic analyzes of these vesicles. This may limit our ability to further investigate which toxins within the OMVs might directly infiltrate the liver. Thirdly, only two-dimensional images of mitochondria were labeled using ImageJ software with the MINA plugin in this study and three-dimensional imaging by employing MitoGraph to provide a more comprehensive visualization of mitochondria will be used in our future study. While our short-term eradication study demonstrates a rapid reversal of key metabolic and molecular parameters, future studies with longer follow-up periods are warranted to determine if eradication can prevent or reverse the progression to more advanced stages of MASLD, including steatohepatitis and fibrosis. In addition, the validation of the WTAP-YTHDF1-GLUT3 axis has thus far been limited to human NASH samples available in public datasets, and has not yet been conducted within our own cohort. Future research endeavors will focus on quantifying WTAP lactylation levels and evaluating the activity of the WTAP-m6A-YTHDF1-GLUT3 axis in liver biopsy samples from patients diagnosed with MASLD, both in the presence and absence of *H. pylori* infection.

In conclusion, this study reveals a novel regulatory mechanism by which *H. pylori* modulates WTAP via lactylation to aggravate MASLD. Therefore, the status of *H. pylori* should be taken into account in MASLD treatment strategies. Furthermore, the WTAP–YTHDF1–GLUT3 axis may be a potentially promising therapeutic target for MASLD progression.

## Supplementary Material

Supplementary material Supplementary_Materials clean

Supplementary material HepG2 STR

Supplementary material Supplementary Tables

Supplementary material L02 STR

## Data Availability

The RNA-seq data and MeRIP-seq data have been deposited in NCBI under SRA accession numbers. All data in this article are available upon reasonable request from the corresponding authors. https://www.ncbi.nlm.nih.gov/geo/query/acc.cgi?acc=GSE301711
