# Supplementary material for: Helicobacter pylori infection aggravates hepatic steatosis by lactylation-driven WTAP-mediated m6A modification
Source: Gut Microbes. 2025 Dec 12;17(1):2599543. doi: 10.1080/19490976.2025.2599543 (PMC12710952; doi:10.1080/19490976.2025.2599543)
Supplement: Supplementary material — Supplementary_Materials clean [file KGMI_A_2599543_SM2372.docx]

**Supplementary Figures**


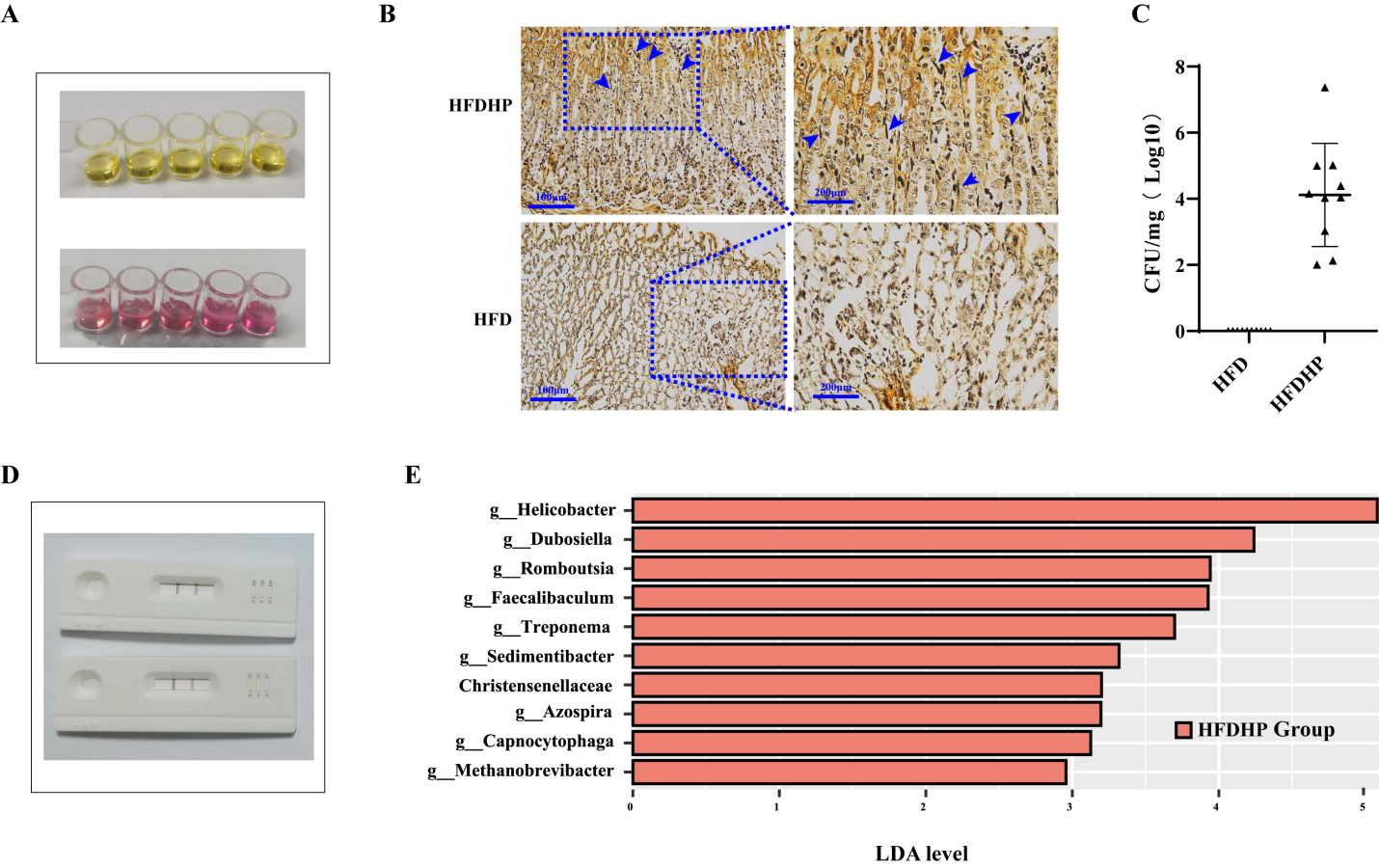


**Figure S1. In vitro and in vivo validation of *H. pylori* successful colonization.**

1. *H. pylori* SS1 strain was cultured in Columbia medium at 37℃ in a three-gas incubator with microaerobic conditions (5% O2, 10% CO2, 75% N2). After 48 hours, the bacteria were washed with Brucella broth, and then the bacteria solution were tested with a positive rapid urease test; (B) Warth-Starry silver staining for gastric tissue in HFD and HFDHP groups. (C) The count of *H. pylori* colonization (CFU/mg) after plating homogenized gastric tissue on WC Dent plates supplemented with 200 g/ml bacitracin, 10 g/ml nalidixic acid and 3 g/ml polymycin B; (D) Mouse fecal antigen detection kit (HpSA, 20183400060, Richen biotech, Taizhou, China) were used and showed positive results, indicating the successful *H. pylori* infection in vivo; (E) The LefsE analysis of differential bacteria and LDA values from microbial 16s rRNA sequencing of gastric tissue. Statistical analysis was performed using Two-tailed Student's *t*-test. **p < 0.05; **p < 0.01;* and ****p < 0.001*.


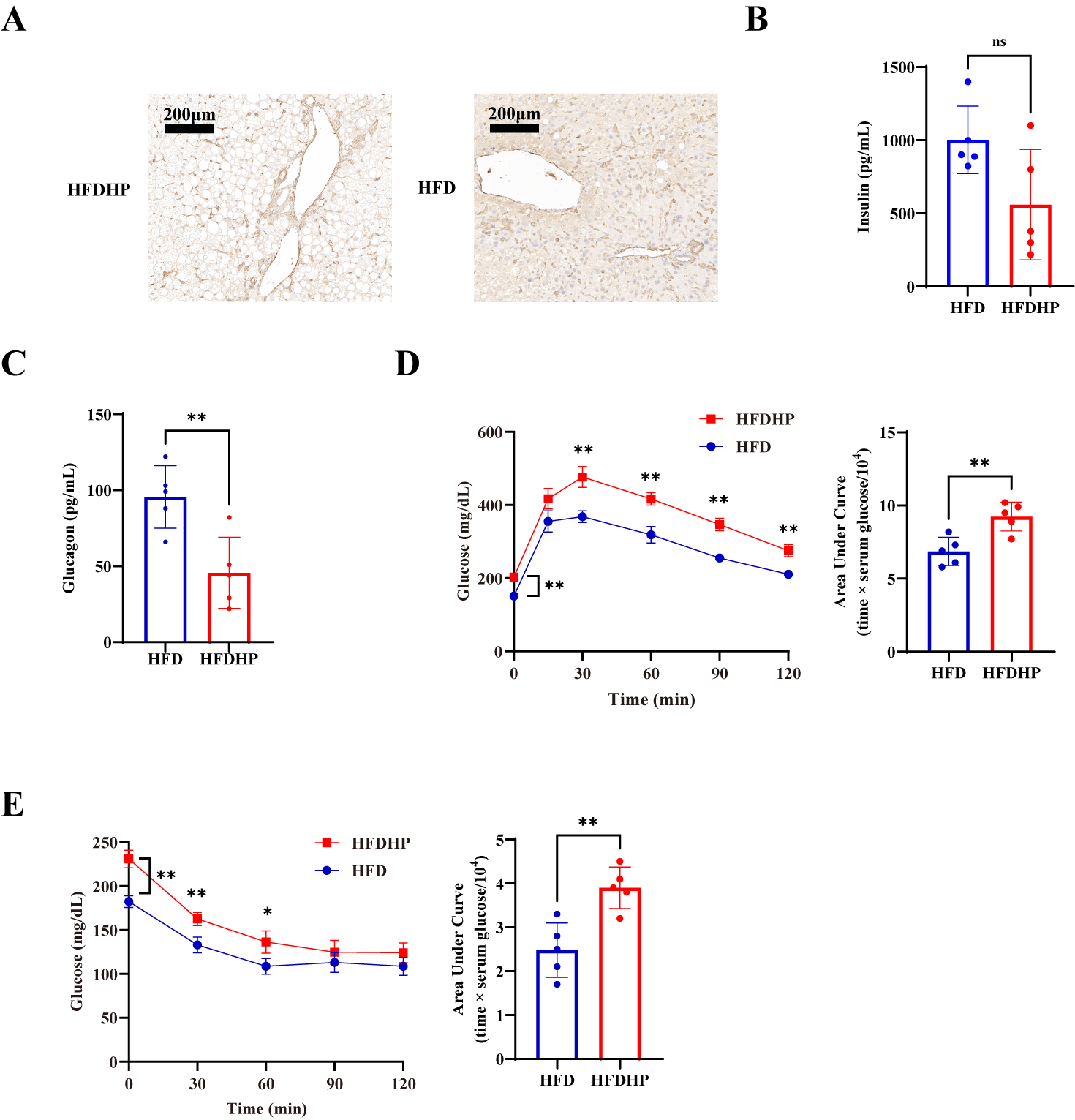


**Figure S2. *H. pylori* infection alters liver glucose metabolism in HFD mice**

1. Representative immunohistochemistry images of α-SMA staining in HFD and HFDHP mice. (B-C) Serum fasting insulin (B) and glucagon (C) levels (pg/mL) of C57/BL6 mice in HFD and HFDHP groups. n = 5 per group; D. Blood glucose levels (mg/mL) and AUC of OGTT test; E. blood glucose levels (mg/mL) of ITT, n = 5 per group. Statistical analysis were performed using Two-tailed Student's *t*-test. **p < 0.05; **p < 0.01;* and ****p < 0.001*.


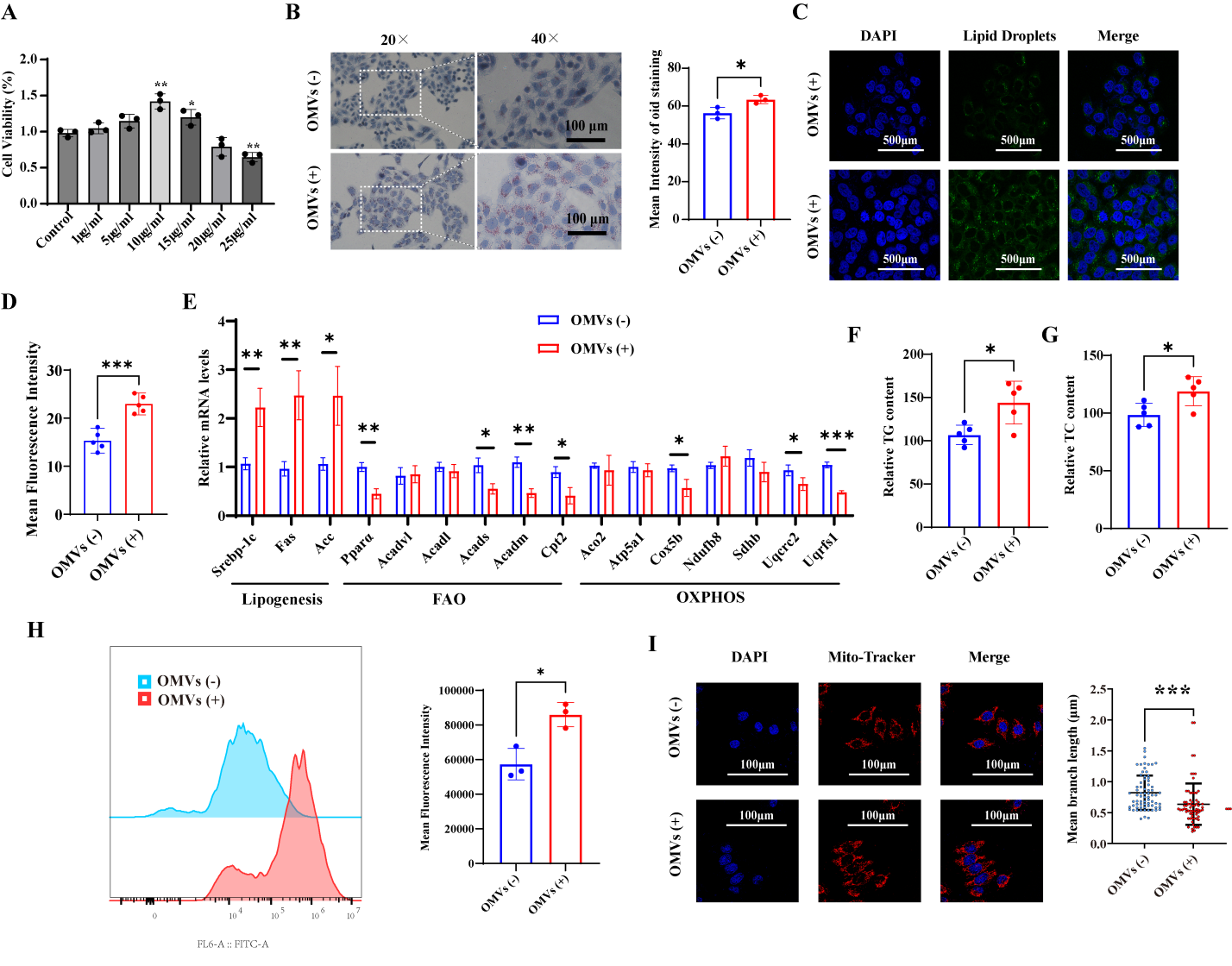


**Figure S3. *H. pylori* OMVs dysregulates lipid metabolism in vitro.**

1. CCK8 analysis of L-02 cells co-culture with OMVs (*H. pylori* 26685 strain) of different concentrations, n = 3 per group; (B) Representative images of Oil Red O (ORO) staining and mean intensity of ORO in L-02 cells with or without OMVs; (C-D) Representative images of immunofluorescence with mean fluorescence intensity of lipid droplets in L-02 cells with or without OMVs; (E) Relative mRNA levels of genes involved in lipogenesis, FAO and OXPHOS using quantitative PCR analysis, n = 3 per group; (F-G) OMVs significantly increases TG (F) and TC (G) contents in L-02, n = 3 per group; (H) Representative histograms and Mean Fluorescence Intensity of ROS production assessed by flow cytometry, n = 3 per group; (I) Representative images of Mito-Tracker staining with mean branch length (μm) of L-02 cells with or without OMVs. Statistical analysis were performed using Student's *t*-test (two groups) and one-way analysis of variance (ANOVA) (multiple groups) followed by Bonferroni's test. **p < 0.05; **p < 0.01;* and ****p < 0.001*.


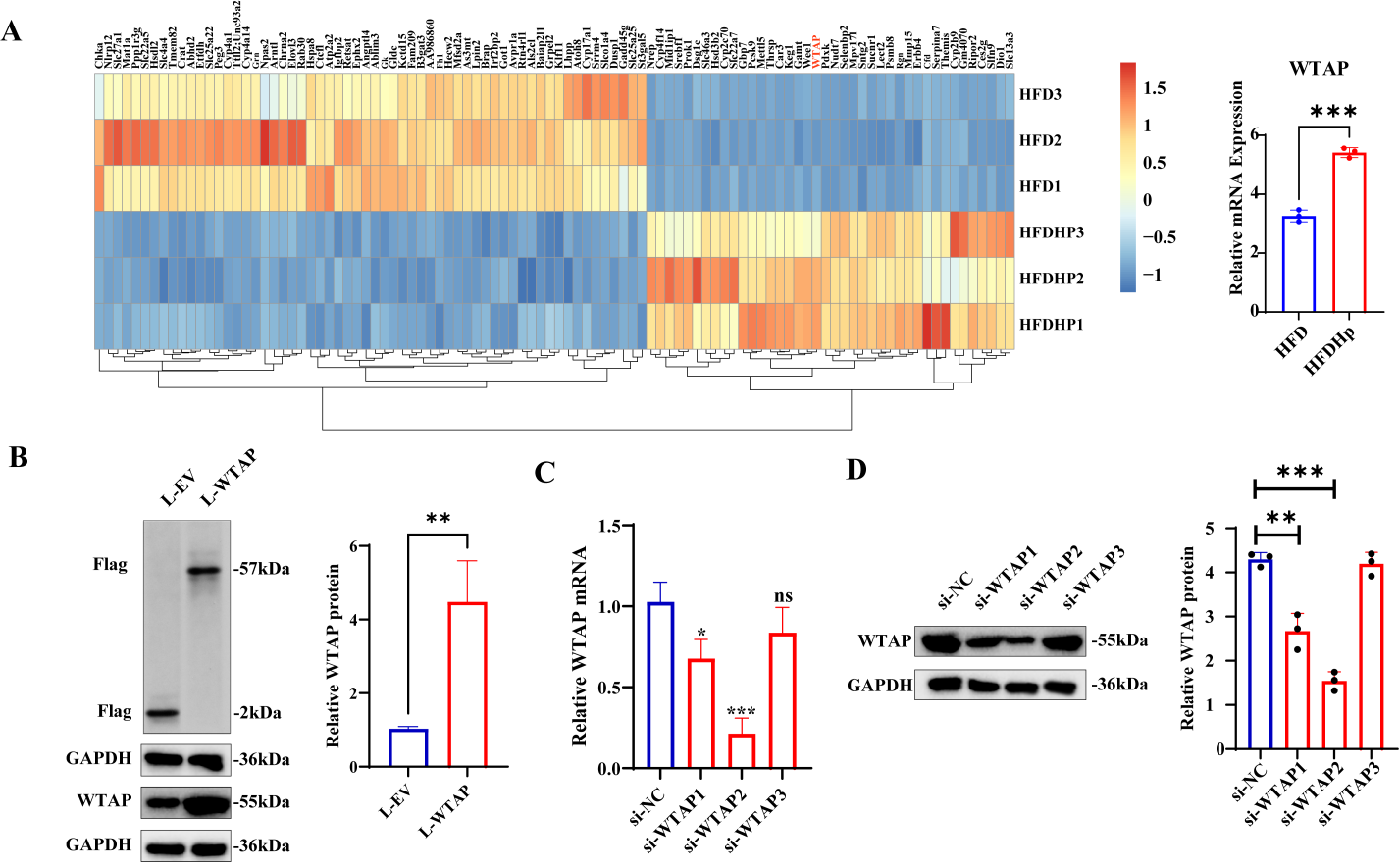


**Figure S4. WTAP expression in vivo and in vitro.**

1. RNA-seq of livers from HFD and HFD mice, n = 5 per group; (B) Western blot confirmed successful transfection of L-WTAP; (C) WTAP mRNA expression after si-WTAP transfection, n = 3 per group; (D) WTAP protein expression after si-WTAP transfection, n = 3 per group. Statistical analysis were performed using Student's *t*-test (two groups) and one-way analysis of variance (ANOVA) (multiple groups) followed by Bonferroni's test. **p < 0.05; **p < 0.01;* and ****p < 0.001*.


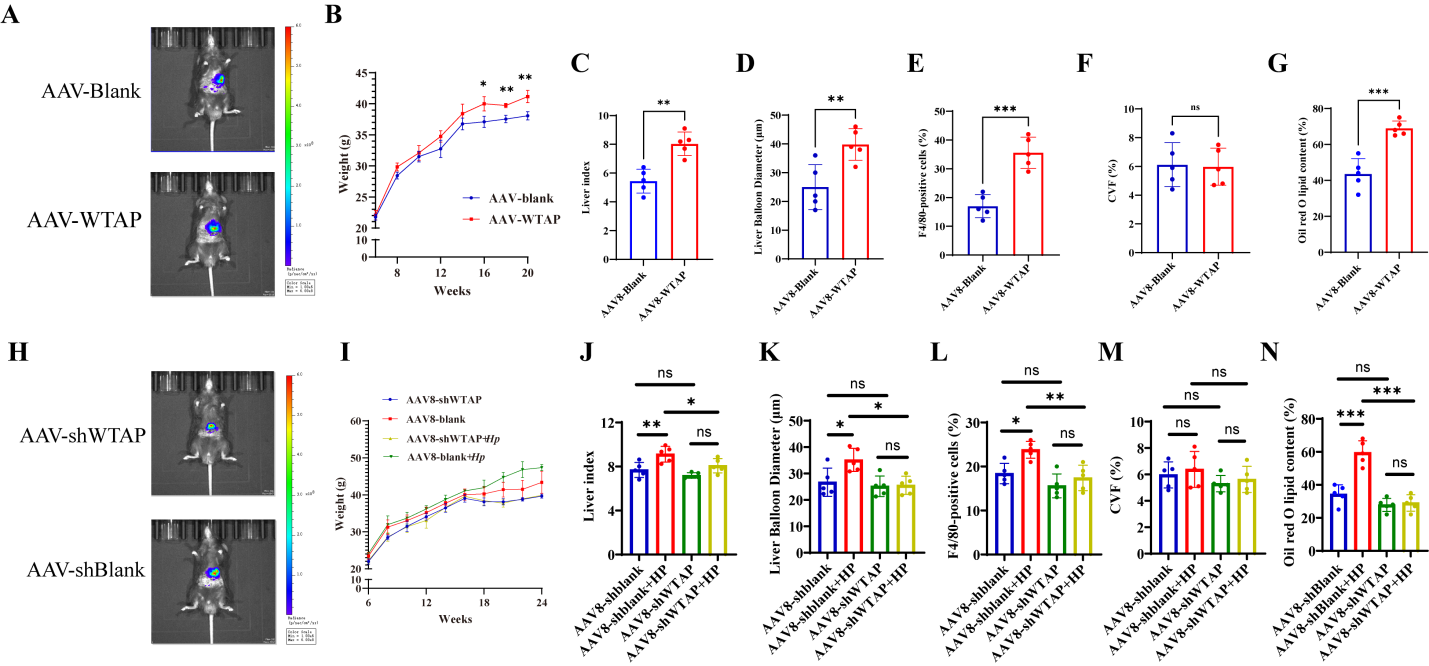


**Figure S5. The role of WTAP in hepatic steatosis in vivo**

(A) Superficial bioluminescence imaging of C57/BL6 mice in which 1×10^8^ AAV8 transduced with WTAP (AAV8-WTAP) and Vehicles (AAV8-Blank) by tail vein injection, respectively. Imaging data was collected using open filters and with an exposure time of 30 s; (B) Weight change of C57/BL6 mice treated with AAV8-WTAP or AAV8-Blank. n = 5 per group; (C-G) Liver index (C), liver balloon diameter (D), number of F4/80 positive cells (E), CVF (F), and oil red lipid content (G) of C57/BL6 mice treated with AAV8-WTAP or AAV8-Blank. n=5 per group; (H) Superficial bioluminescence imaging of C57/BL6 mice in which 1×10^8^ AAV8 transduced with shWTAP (AAV8-shWTAP) and Vehicles (AAV8-shBlank) by tail vein injection, respectively; (I) Weight change of C57/BL6 mice treated with AAV8-shWTAP, AAV8-shWTAP+*H. pylori* infection, AAV8-shBlank, and AAV8-shWTAP+*H. pylori* infection. n=5 per group; (J-N) Liver index (J), liver balloon diameter (K), number of F4/80 positive cells (L), CVF (M), and oil red lipid content (N) of C57/BL6 mice treated with AAV8-WTAP or AAV8-Blank. n=5 per group. Statistical analysis were performed using Student's *t*-test (two groups) and one-way analysis of variance (ANOVA) (multiple groups) followed by Bonferroni's test. **p < 0.05; **p < 0.01;* and ****p < 0.001*.


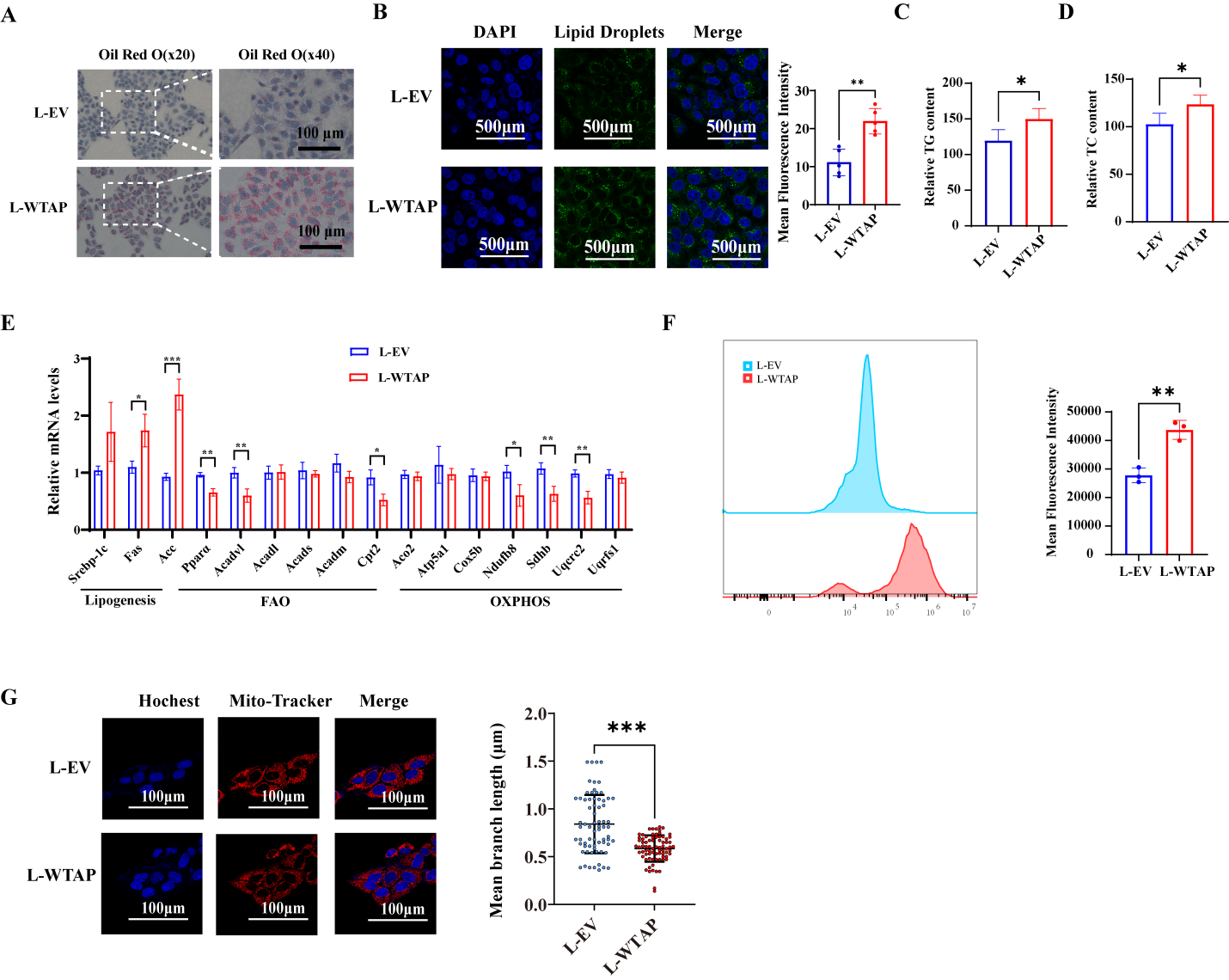


**Figure S6. WTAP overexpression dysregulates lipid metabolism in L-02 cells**

(A) Representative images of Oil red O staining of L-02 cells transduced with L-EV or L-WTAP incubated with 30 μM BSA-conjugated-PA for 24 hours; (B) Representative fluorescence images and mean fluorescence intensity of HepG2 cells transfected with L-EV or L-WTAP with LDs visualized using BODIPY 493/503 staining; (C-D) Intracellular TG (C) or TC (D) in L-02 cells transfected with L-EV or L-WTAP, n=3 per group; (E) Relative mRNA levels of genes involved in lipogenesis, FAO and OXPHOS using quantitative PCR analysis, n = 3 per group; (F) Intracellular ROS levels of L-02 cells transfected with L-EV or L-WTAP by flow cytometry; (G) Representative confocal images of Mitochondrial Tracker Red in L-02 cells transfected with L-EV or L-WTAP. Statistical analysis were performed using Student's *t*-test (two groups) and one-way analysis of variance (ANOVA) (multiple groups) followed by Bonferroni's test. **p < 0.05; **p < 0.01;* and ****p < 0.001*.


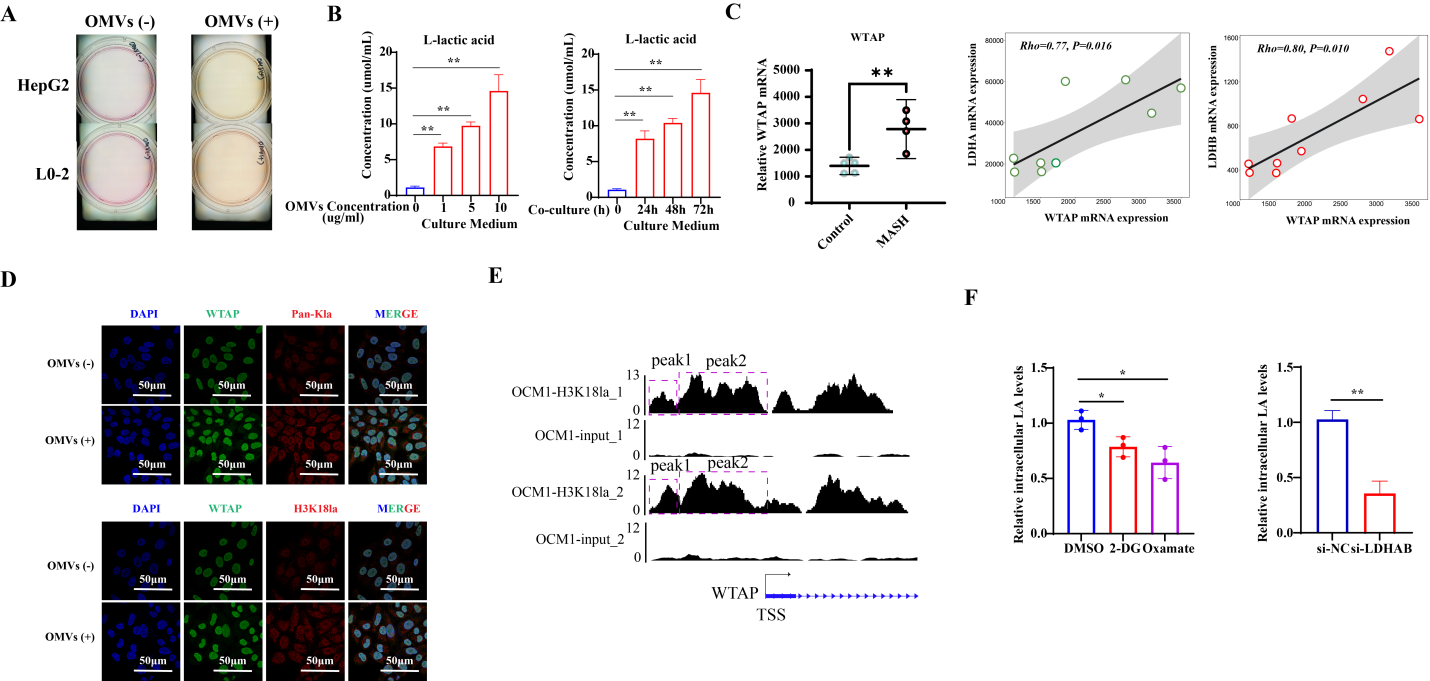


**Figure S7. *H. pylori*** **infection promotes glycolysis in liver.**

(A) The color of cell culture medium from liver cells with or without OMVs co-culture; (B) LA concentration in culture medium co-culture with OMVs at indicated concentrations or time, n=3 per group; (C) Public dataset (GSE260666) suggested that WTAP expression is higher in MASH patients and positively correlated with glycolysis-related genes (LDHA and LDHB); (D) Immunofluorescence of WTAP, Pan-Kla and H3K18la in HepG2 cells with or without OMVs; (E) Enrichment of the H3K18la signal (two peaks) in the promoter region of the FTO gene is demonstrated by ChIP-Seq using anti-H3K18la antibodies (GEO accession number: GSE156675); (F) Intracellular LA levels after cells treated with 2-DG or oxamate and cells transfected with si-LDHAB, n=5 per group. Statistical analysis were performed using Student's *t*-test (two groups) and one-way analysis of variance (ANOVA) (multiple groups) followed by Bonferroni's test. **p < 0.05; **p < 0.01;* and ****p < 0.001*.

**
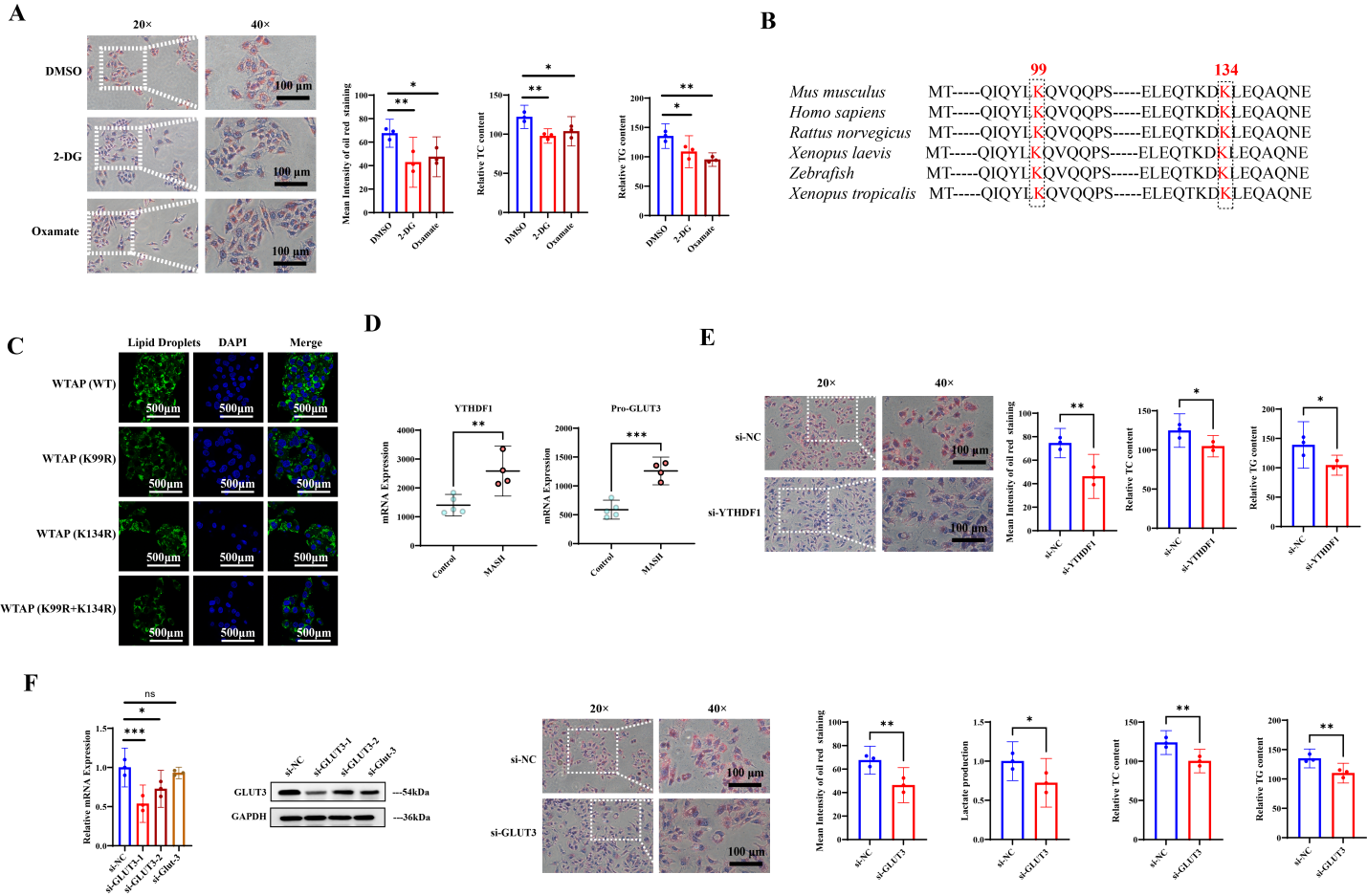
**

**Figure S8.** **The glycolytic-WTAP–YTHDF1–GLUT3 axis in *H. pylori*-mediated MASLD.**

(A) Representative images and mean intensity of ORO staining of HepG2 cells upon inhibiting glycolysis through the application of 2-DG and oxamate; (B) WTAP sequence alignment in different species (*Mus musculus*, Homo *sapiens*, *Rattus norvegicus*, *Xenopus laevis*, *Zebrafish* and *Xenopus tropicalis*) conservation analysis of potential lactylation modification sequence sites for WTAP; (C) Fluorescence microscopy images of HepG2 cells transfected with Flag-WTAP (WT), Flag-WTAP (K99R), Flag-WTAP (K134R) and Flag-WTAP (K99R+K134R) with LDs visualized using BODIPY 493/503 staining; The green signals represent PKH67 labeled OMVs, while the blue represents DAPI. (D) Public dataset (GSE260666) suggested that YTHDF1 and pro-GLUT3 expression is higher in MASH patients compared with healthy controls. (E) Representative images and mean intensity of ORO staining of HepG2 cells in si-NC and si-YTHDF1 groups. (F) GLUT3 mRNA levels and protein expression detected by qPCR and western blotting in HepG2 cells transfected with distinct siRNAs. (G) Representative images and mean intensity of ORO staining of HepG2 cells in si-NC and si-GLUT3 groups, n = 3 per group. Statistical analysis were performed using Student's *t*-test (two groups) and one-way analysis of variance (ANOVA) (multiple groups) followed by Bonferroni's test. **p < 0.05; **p < 0.01;* and ****p < 0.001*.

**
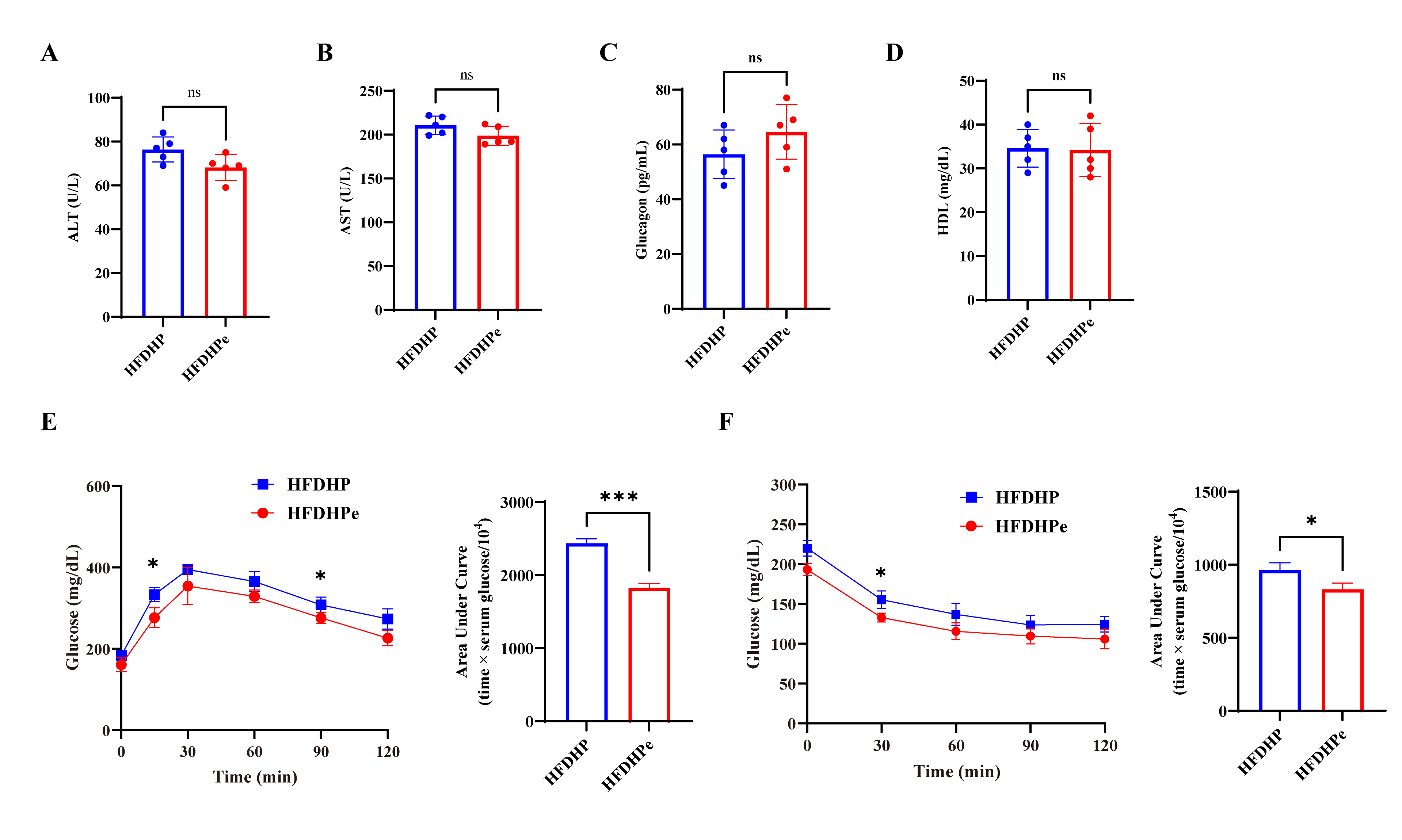
**

**Figure S9. *H. pylori*** **infection eradication alleviates hepatic steatosis in HFDHP mice.**

(A-D) Serum concentration of ALT (U/L) (A), AST (U/L) (B), glucagon (pg/mL) (C), and HDL (mg/dl) (D) in C57/BL6 mice in *H. pylori*-infected C57/BL6 mice eradicated with triple therapy or placebo. n=5 per group; (E) Blood glucose levels (mg/mL) and AUC of OGTT; (F) Blood glucose levels (mg/mL) of ITT, n=5 per group; Statistical analysis were performed using Student's *t*-test. **p < 0.05; **p < 0.01;* and ****p < 0.001*.

**Supplementary Tables**

| **Gene names** | **Sequences (5'-3')** |
| --- | --- |
| Mouse-β-actin-Forward | CAGCCATGTACGTTGCTATCCAGG |
| Mouse-β-actin-Reverse | AGGTCCAGACGCAGGATGGCATG |
| Mouse-LDHA-Forward | TATCTTAATGAAGGACTTGGCGGATGAG |
| Mouse-LDHA-Reverse | GGAGTTCGCAGTTACACAGTAGTC |
| Human-WTAP-Forward | GGCGAAGTGTCGAATGCT |
| Human-WTAP-Reverse | CCAACTGCTGGCGTGTCT |
| Mouse-Mettl3-Forward | CTGGGCACTTGGATTTAAGGAA |
| Mouse-Mettl3-Reverse | TGAGAGGTGGTGTAGCAACTT |
| Mouse-Mettl5-Forward | GAACTAGAGAGTCGCCTGCAA |
| Mouse-Mettl5-Reverse | CTGCAACCGCTTTGTTTTCAA |
| Mouse-Mettl14-Forward | GACTGGCATCACTGCGAATGA |
| Mouse-Mettl14-Reverse | AGGTCCAATCCTTCCCCAGAA |
| Mouse-WTAP-Forward | GGAAGTTTACGCCTGATAGCC |
| Mouse-WTAP-Reverse | TCTGCTTCAAGCTGTGCAAT |
| Mouse-ALKBH5-Forward | TTGCCACCCAGCTATGCTTC |
| Mouse-ALKBH5-Reverse | CAGACCGCCGGTTTTCTTCTT |
| Mouse-FTO-Forward | TTCATGCTGGATGACCTCAATG |
| Mouse-FTO-Reverse | GCCAACTGACAGCGTTCTAAG |
| Human-YTHDF1-Forward | CAAGCACACAACCTCCATCTTCG |
| Human-YTHDF1-Reverse | GTAAGAAACTGGTTCGCCCTCAT |
| Human-GLUT3-Forward | AGCTCTCTGGGATCAATGCTGTGT |
| Human-GLUT3-Reverse | ATGGTGGCATAGATGGGCTCTTGA |
| Mouse-GLUT3-Forward | AGCTCTCTGGGATCAATGCTGTGT |
| Mouse-GLUT3-Reverse | ATGGTGGCATAGATGGGCTCTTGA |
| Human-Uqrfs1-Forward | CCTGTGTTGGACCTGAAGC |
| Human-Uqrfs1-Reverse | ATAACAAACAGAAGCAGGGACAT |
| Human-Uqcrc2-Forward | GTTTGTTCATTAAAGCAGGCAGTAG |
| Human-Uqcrc2-Reverse | TGCTTCAATTCCACGGGTTATC |
| Human-Acadvl-Forward | TAGGAGAGGCAGGCAAACAGCT |
| Human-Acadvl-Reverse | CACAGTGGCAAACTGCTCCAGA |
| Human-Acadl-Forward | GTTTGGACTCCGCCACTGCTTG |
| Human-Acadl-Reverse | GGCTGAACTCTGGCATCCACAT |
| Human-Acadm-Forward | TAATCGGTGAAGGAGCAGGTTT |
| Human-Acadm-Reverse | GGCATACTTCGTGGCTTCGT |
| Human-Ndufb8-Forward | CTCCTTGTTGGGCTTATCACA |
| Human-Ndufb8-Reverse | GCCCACTCTAGAGGAGCTGA |
| Human-Acads-Forward | TTACCTGGCCTACTCCATCG |
| Human-Acads-Reverse | TGATCCACTGTTGCTTCTGC |
| Human-ATP5A1-Forward | ACTGGGCGTGTCTTAAGTATTG |
| Human-ATP5A1-Reverse | ACCAAGGGCATCAACTACAC |
| Human-Aco2-Forward | AGCCCAACGAGTACATCCAT |
| Human-Aco2-Reverse | TCTTCTCCGAGAGTGTCAGC |
| Human-Cpt2-Forward | GAAGAAGCTGAGCCCTGATG |
| Human-Cpt2-Reverse | GCCATGGTATTTGGAGCACT |
| Human-COX5B-Forward | AGGCAGCTTCAGGCACCAAG |
| Human-COX5B-Reverse | GGTGGGGCACCAGCTTGTAA |
| Human-Sdhb-Forward | GGCGGAAGGTGGTTGGCTGA |
| Human-Sdhb-Reverse | AGCTCAGGCCCTGGCGCTCT |
| Human-Srebp-1c-Forward | GCAGCCACCATCTAGCCTG |
| Human-Srebp-1c-Reverse | CAGCAGTGAGTCTGCCTTGAT |
| Human-Acc-Forward | ATGGGCGGAATGGTCTCTTTC |
| Human-Acc-Reverse | TGGGGACCTTGTCTTCATCAT |
| Human-Fas-Forward | CACAGGGACAACCTGGAGTT |
| Human-Fas-Reverse | ACTCCACAGGTGGGAACAAG |
| Human-Pparα-Forward | GGACCTTCGGCAGCTGGT |
| Human-Pparα-Reverse | TCGGACTCGGTCTTCTTGATG |
| Mouse-Pparα-Forward | AACATCGAGTGTCGAATATGTGG |
| Mouse-Pparα-Reverse | CCGAATAGTTCGCCGAAAGAA |
| Mouse-Fas-Forward | CTTGGGTGCTGACTACAACC |
| Mouse-Fas-Reverse | GCCCTCCCGTACACTCACTC |
| Mouse-Acc-Forward | AGGAAGATGGCGTCCGCTCTG |
| Mouse-Acc-Reverse | GGTGAGATGTGCTGGGTCAT |
| Mouse-Srebp-1c-Forward | GGAGCCATGGATTGCACATT |
| Mouse-Srebp-1c-Reverse | GGCCAGGGAAGTCACTGT |
| Mouse-Acadvl-Forward | ACTACTGTGCTTCAGGGACAA |
| Mouse-Acadvl-Reverse | GCAAAGGACTTCGATTCTGCC |
| Mouse-Acadl-Forward | TTTCCTCGGAGCATGACATTTT |
| Mouse-Acadl-Reverse | GCCAGCTTTTTCCCAGACCT |
| Mouse-Acads-Forward | GACTGGCGACGGTTACACA |
| Mouse-Acads-Reverse | GGCAAAGTCACGGCATGTC |
| Mouse-Acadm-Forward | TGACGGAGCAGCCAATGA |
| Mouse-Acadm-Reverse | TCGTCACCCTTCTTCTCTGCTT |
| Mouse-Cpt2-Forward | CAGCACAGCATCGTACCCA |
| Mouse-Cpt2-Reverse | TCCCAATGCCGTTCTCAAAAT |
| Mouse-Ndufb8-Forward | TGTTGCCGGGGTCATATCCTA |
| Mouse-Ndufb8-Reverse | AGCATCGGGTAGTCGCCATA |
| Mouse-Sdhb-Forward | CTGAATAAGTGCGGACCTATGG |
| Mouse-Sdhb-Reverse | AGTATTGCCTCCGTTGATGTTC |
| Mouse-Uqrfs1-Forward | GGTAACTGCAACTACTACTGTGG |
| Mouse-Uqrfs1-Reverse | CTTGATCTCGATCTTCGACATGG |
| Mouse-Uqcrc2-Forward | AAAGTTGCCCCGAAGGTTAAA |
| Mouse-Uqcrc2-Reverse | GAGCATAGTTTTCCAGAGAAGCA |
| Mouse-Aco2-Forward | ATCGAGCGGGGAAAGACATAC |
| Mouse-Aco2-Reverse | TGATGGTACAGCCACCTTAGG |
| Mouse-Atp5a1-Forward | TCTCCATGCCTCTAACACTCG |
| Mouse-Atp5a1-Reverse | CCAGGTCAACAGACGTGTCAG |
| Mouse-Cox5b-Forward | GCTGCATCTGTGAAGAGGACAAC |
| Mouse-Cox5b-Reverse | CAGCTTGTAATGGGTTCCACAGT |
| Mouse-Ldhb-Forward | CATTGCGTCCGTTGCAGATG |
| Mouse-Ldhb-Reverse | GGAGGAACAAGCTCCCGTG |
| Human-β-actin-Forward | AAGTGTGACGTTGACATCCG |
| Human-β-actin-Reverse | GATCCACATCTGCTGGAAGG |
| Human-METTL5-Forward | CTTTGGAAATGGCAAGAACAGCAG |
| Human-METTL5-Reverse | ATTAGGTCCACTTCAATGTCCACTG |
| Mouse-LDHB-Forward | CATTGCGTCCGTTGCAGATG |
| Mouse-LDHB-Reverse | GGAGGAACAAGCTCCCGTG |
| Human-LDHA-Forward | AGCTTCCATTTAAGGCCCCG |
| Human-LDHA-Reverse | TCTTTTGAGACCGCTAGTGC |
| Human-LDHB-Forward | TTCTGCTAGATTTCGCTACC |
| Human-LDHB-Reverse | TTCATTCTCAATGCCATACA |
| Human-METTL14-Forward | GAACACAGAGCTTAAATCCCCA |
| Human-METTL14-Reverse | TGTCAGCTAAACCTACATCCCTG |
| Human-WTAP-Forward | GGCGAAGTGTCGAATGCT |
| Human-WTAP-Reverse | CCAACTGCTGGCGTGTCT |
| Human-ALKBH5-Forward | CCCGAGGGCTTCGTCAACA |
| Human-ALKBH5-Reverse | CGACACCCGAATAGGCTTGA |
| Human-FTO-Forward | GCCTCGGTTTAGTTCCACTCAC |
| Human-FTO-Reverse | GTCGCCATCGTCTGAGTCATTG |
| Human-METTL3-Forward | AAGGAGCCGGCTAAGAAGTC |
| Human-METTL3-Reverse | TCACTGGCTTTCATGCACTC |
| Human-GLUT14-Forward | CTGCTCACGAATCTCTGGTCC |
| Human-GLUT14-Reverse | GCCTAATAGCACCGGCCATAG |
| siRNA-human-WTAP-1 | CAAGAGAUGCAAGAGUGUACU |
| siRNA-human-WTAP-2 | GGAACAGACUAAAGACAAACU |
| siRNA-human-WTAP-3 | AGAGAUGAGUUAAUUCUAAGA |
| siRNA-human-YTHDF1-1 | GGAGAAUAACGACAACAAACC |
| siRNA-human-YTHDF1-2 | GGACAGUCAAAUCAGAGUAAC |
| siRNA-human-YTHDF1-3 | GGCGUGUGUUCAUCAUCAAGA |
| siRNA-negative control | sense: UUCUCCGAACGUGUCACGUdTdT |
|  | antisense: ACGUGACACGUUCGGAGAAdTdT |
| GLUT3-1915-F | CCCCTCCGCTGCTCACTAT |
| GLUT3-1915-R | CGTGTGCCTGCCCTTCAA |
| GLUT3-2116-F | TAAGTCGTGCCTCCTTCCAC |
| GLUT3-2116-R | GTAGCAGCATTCAGAAGCGT |
| WTAP^MU^ F | TGGAAGTTTAGTACCAGCAGGACTACAGC |
| WTAP^MU^ R | CTGGTACTAAACTTCCAGGCACTCAGTT |
| WTAP^WT^ F | 5ACCGGACTCAGATCTCGAGCCACCATGACCAACGAAGAACCTC |
| WTAP^WT^ R | ATCTAGAGTCGCGGGATCTTACAAAACTGAACCCTGTACATT |

Table S1. Primer information for gene amplification and siRNA sequences for gene silencing.

| **Antigen** | **Manufacturer** | **Catalog Number** | **Application** |
| --- | --- | --- | --- |
| METTL5 | Proteintech (Wuhan, Hubei, China) | 16791-1-AP | 1:1000 for WB |
| WTAP | Proteintech | 10200-1-AP | 1:1000 for WB;  1:200 for IF;  1:100 for IP;  1:50 for RIP |
| METTL3 | Proteintech | 15073-1-AP | 1:1000 for WB |
| FTO | Proteintech | 27226-1-AP | 1:1000 for WB |
| ALKBH5 | Proteintech | 16837-1-AP | 1:1000 for WB |
| Flag | Proteintech | 20543-1-AP | 1:1000 for IP |
| Lamin A/C | Proteintech | 10298-1-AP | 1:5000 for WB |
| YTHDF1 | Proteintech | 17479-1-AP | 1:1000 for WB |
| CagA | Santa Cruz Biotechnology (Dallas, TX, USA) | sc-25766 | 1:3000 for WB |
| UreaA | Santa Cruz Biotechnology | sc-21016 | 1:3000 for WB |
| Pan-Kla | PTM-bio (Hangzhou, Zhejiang, China) | PTM-1401RM | 1:1000 for WB |
| H3K9la | PTM-bio | PTM-1419RM | 1:1000 for WB |
| H3K14la | PTM-bio | PTM-1414RM | 1:1000 for WB |
| H3K18la | PTM-bio | PTM-1427RM | 1:1000 for WB |
| H3K27la | PTM-bio | PTM-1428 | 1:1000 for WB |
| H3K56la | PTM-bio | PTM-1421RM | 1:1000 for WB |
| Histone-H3 | PTM-bio | PTM-1001RM | 1:5000 for WB |
| CD63 | Proteintech | 25682-1-AP | 1:1000 for WB |
| CD9 | Proteintech | 20597-1-AP | 1:1000 for WB |
| Alix | Proteintech | 12422-1-AP | 1:10000 for WB |
| Calnexin | Proteintech | 10427-2-AP | 1:10000 for WB |
| GLUT3 | Proteintech | 20403-1-AP | 1:1000 for WB |
| Myc | Proteintech | 60003-2-Ig | 1:500 for IF |
| GAPDH | Proteintech | 60004-1-Ig | 1:5000 for WB |
| LDHA | Proteintech | 21799-1-AP | 1:1000 for WB |
| LDHB | Proteintech | 14824-1-AP | 1:1000 for WB |
| β-actin | Proteintech | 66009-1-Ig | 1:5000 for WB |
| N6-methyladenosine | Abcam | EPR23561-164 | 1 ug antibody/100 ug RNA, MeRIP-Seq;  1:1000, dot blot;  1:100, MeRIP-qPCR |
| VacA | Austral Biologicals (San Ramon, CA, USA) | HPP-5013-9 | 1:3000 for WB |
| IgG | Servicebio (Wuhan, Hubei, China) | GB23301 | IP |
| Goat Anti-Mouse IgG-Fc Secondary Antibody (HRP) | Proteintech | SA00001-1 | 1:10000 for WB |
| Goat Anti-Rabbit IgG-Fc Secondary Antibody (HRP) | Proteintech | SA00001-2 | 1:10000 for WB |
| CoraLite488-conjugated Goat Anti-Rabbit IgG(H+L) | Proteintech | SA00013-2 | 1:200 for IF |
| CoraLite488-conjugated Goat Anti-Mouse IgG(H+L) | Proteintech | SA00013-1 | 1:200 for IF |
| CoraLite594-conjugated Goat Anti-Rabbit IgG | Proteintech | SA00013-4 | 1:200 for IF |
| CoraLite594-conjugated Goat Anti-Mouse IgG | Proteintech | SA00013-3 | 1:200 for IF |

Table S2. Primary antibodies and secondary antibodies used in this study.

**Table S3. Detailed information of m^6^A-Peaks detected by MeRIP-seq analysis.**

**Table S4. Detailed information of mRNA enrichment according to mRNA-seq analysis.**

**Table S5. The overlapping genes significantly changed in MeRIP-seq and mRNA-seq analysis.**
