# Supplementary material for: Helicobacter pylori infection aggravates hepatic steatosis by lactylation-driven WTAP-mediated m6A modification
Source: Gut Microbes. 2025 Dec 12;17(1):2599543. doi: 10.1080/19490976.2025.2599543 (PMC12710952; doi:10.1080/19490976.2025.2599543)

Hep-G2 Cell STR Identification

● Experimental Procedure

Utilize Vazyme Cell Genomic DNA Extraction Kit to extract DNA from the sample. Employ the Yuewei Gene Human STR locus detection kit to amplify the sample. Collect signals using the Applied Biosystems SeqStudio Genetic Analyzer. Analyze the loci using Genemapper software 6.

Prepare the reaction mixture in a PCR tube according to the following system:

**Components**	**Volume (Content)**
PCR Master Mix	5.0 μl
Primer Mix	2.5 μl
Sample	30 ng
DEPC Water	To 10.0 μl

Perform the amplification reaction in a PCR instrument with the following program:

**Temperature**	**Time**	**Cycles**
95°C	5 min	N/A
94°C	10 s	28-30 cycles
61°C	1 min	
70°C	30 s	
60°C	15 min	N/A

Prepare the electrophoresis sample in the PCR tube: Denature at 95°C for 3 min → Cool down to 4°C for 3 min, and then collect data using the machine.

Components	Volume
Hi-Di™ Formamide	8.5 μl
SIZE	0.5 μl
Sample	1.0 μl

● Experimental Results

Sample ID	Database	Matched Cell	Matching Rate	Description
Hep-G2	ATCC, DSMZ	Hep-G2	98.5%	Matched

Analysis of Sample Loci Report Values

Loci	Hep-G2 Sample STR Report Value			Hep-G2 Database Reference Value		
	AL1	AL2	AL3	AL1	AL2	AL3
**Amel**	X	Y		X	Y	
**D3S1358**	15	16		15	16	
**TH01**	9			9		
**D21S11**	29	31		29	31	
**D18S51**	13	14		13	14	
**Penta E**	15	20		15	20	
**D5S818**	11	12		11	12	
**D13S317**	9	13		9	13	
**D7S820**	10			10		
**D16S539**	12			12	13	
**CSF1PO**	10	11		10	11	
**Penta D**	9	13		9	13	
**vWA**	17			17		
**D8S1179**	15	16		15	16	
**TPOX**	8	9		8	9	
**FGA**	22	25		22	25	
**D19S433**	15.2			15.2		
**D12S391**	21	25		21	25	
**D6S1043**	13					
**D2S1338**	19	20		19	20	
**D1S1656**	11	12		11	12	

Sample STR peak map

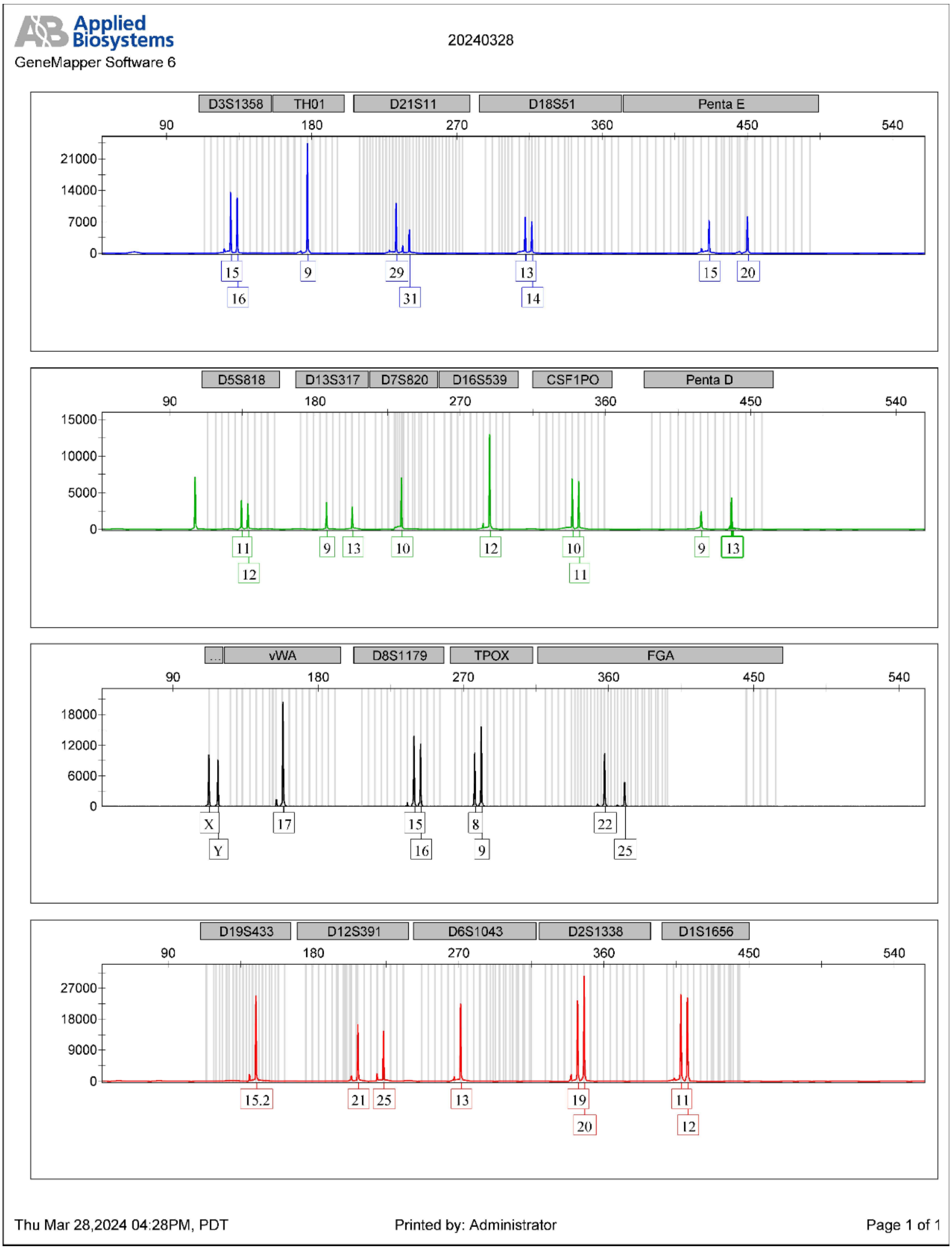

Supplement: Supplementary material — HepG2 STR [file KGMI_A_2599543_SM2373.pdf]
